# Contralesional Cathodal Transcranial Direct Current Stimulation Does Not Enhance Upper Limb Function in Subacute Stroke: A Pilot Randomized Clinical Trial

**DOI:** 10.1155/2021/8858394

**Published:** 2021-08-10

**Authors:** Danielle De S. Boasquevisque, Larissa Servinsckins, Joselisa P. Q. de Paiva, Daniel G. dos Santos, Priscila Soares, Danielle S. Pires, Jed A. Meltzer, Ela B. Plow, Paloma F. de Freitas, Danielli S. Speciali, Priscila Lopes, Mario F. P. Peres, Gisele S. Silva, Shirley Lacerda, Adriana B. Conforto

**Affiliations:** ^1^Hospital Israelita Albert Einstein, São Paulo 05652-900, Brazil; ^2^Population Health Research Institute, Hamilton, Canada L8L 2X2; ^3^Rotman Research Institute, Baycrest Centre, Toronto, Canada M6A2E; ^4^Cleveland Clinic Foundation, Cleveland 44195, USA; ^5^Federal University of São Paulo, São Paulo 04039-000, Brazil; ^6^Hospital das Clínicas, São Paulo University, São Paulo 05403-000, Brazil

## Abstract

Transcranial direct current stimulation (tDCS) has the potential to improve upper limb motor outcomes after stroke. According to the assumption of interhemispheric inhibition, excessive inhibition from the motor cortex of the unaffected hemisphere to the motor cortex of the affected hemisphere may worsen upper limb motor recovery after stroke. We evaluated the effects of active cathodal tDCS of the primary motor cortex of the unaffected hemisphere (ctDCSM1_UH_) compared to sham, in subjects within 72 hours to 6 weeks post ischemic stroke. Cathodal tDCS was intended to inhibit the motor cortex of the unaffected hemisphere and hence decrease the inhibition from the unaffected to the affected hemisphere and enhance motor recovery. We hypothesized that motor recovery would be greater in the active than in the sham group. In addition, greater motor recovery in the active group might be associated with bigger improvements in measures in activity and participation in the active than in the sham group. We also explored, for the first time, changes in cognition and sleep after ctDCSM1_UH_. Thirty subjects were randomized to six sessions of either active or sham ctDCSM1_UH_ as add-on interventions to rehabilitation. The NIH Stroke Scale (NIHSS), Fugl-Meyer Assessment of Motor Recovery after Stroke (FMA), Barthel Index (BI), Stroke Impact Scale (SIS), and Montreal Cognitive Assessment (MoCA) were assessed before, after treatment, and three months later. In the intent-to-treat (ITT) analysis, there were significant GROUP∗TIME interactions reflecting stronger gains in the sham group for scores in NIHSS, FMA, BI, MoCA, and four SIS domains. At three months post intervention, the sham group improved significantly compared to posttreatment in FMA, NIHSS, BI, and three SIS domains while no significant changes occurred in the active group. Also at three months, NIHSS improved significantly in the sham group and worsened significantly in the active group. FMA scores at baseline were higher in the active than in the sham group. After adjustment of analysis according to baseline scores, the between-group differences in FMA changes were no longer statistically significant. Finally, none of the between-group differences in changes in outcomes after treatment were considered clinically relevant. In conclusion, active CtDCSM1_UH_ did not have beneficial effects, compared to sham. These results were consistent with other studies that applied comparable tDCS intensities/current densities or treated subjects with severe upper limb motor impairments during the first weeks post stroke. Dose-finding studies early after stroke are necessary before planning larger clinical trials.

## 1. Introduction

Stroke is a leading cause of disability worldwide. Hand paresis affects up to 80% of the subjects in the acute phase after ischemic stroke and substantially contributes to disability [1, 2]. Over the past several decades, transcranial direct current stimulation (tDCS) has emerged as a potential tool to enhance upper limb motor recovery [3–9].

The motor cortex of the unaffected hemisphere (M1_UH_) may have a maladaptive role in motor recovery by overinhibition of the motor cortex of the affected hemisphere (M1_AH_) according to the theory of interhemispheric inhibition [10]. Cathodal tDCS to inhibit M1_UH_ (ctDCSM1_UH_) and hence disinhibit M1_AH_ has been investigated as a potential add-on therapy to upper limb rehabilitation. Until now, there is limited information about the effects of ctDCSM1_UH_ during the first weeks after stroke when mechanisms of neuroplasticity are more active. Effective rehabilitation strategies delivered in this early phase are deemed pivotal to enhance recovery [11–17]. Meta-analyses concluded that ctDCSM1_UH_ may be beneficial for improvement of upper limb function when delivered in the chronic phase, but not at earlier stages after stroke [8, 18]. However, most of the research included subjects in chronic than in early stages.

Only five studies focused on the effects of ctDCSM1_UH_ in the subacute phase after stroke [12, 13, 15, 16]. In the acute phase, up to seven days after stroke according to the definition of the Stroke Recovery and Rehabilitation Roundtable taskforce [19], two studies assessed the effects of tDCS. In summary, the time of stroke onset varied from less than 10 days to less than 10 weeks; the numbers of treatment sessions were 2, 6, 9, 10, 15, or 30; treatment was administered on consecutive days in most studies except for one [16]; current intensities were 1, 1.5, or 2 mA with estimated current densities varying from 0.029 to 0.08 mA/cm^2^. In regard to timing (before, during, or after other rehabilitation intervention), two out of seven trials delivered tDCS before therapy [12, 13], four during therapy [13, 15–17], and one did not include any therapy [20]. Rehabilitation interventions were very diverse, including physical therapy, occupational therapy, robot-aided therapy, or motor practice.

In addition to the paucity of data and the variety of paradigms in the few studies that addressed the effects of ctDCS_UH_ in the subacute stage, a systematic review concluded that there is limited information about adverse events of tDCS in subjects post stroke [21].

The main objective of this study was to assess safety. Our primary findings, published elsewhere, showed that the active intervention was safe, compared to sham [22]. We also collected preliminary data regarding efficacy to inform plans for larger trials.

We hypothesized that motor recovery would be greater in the active than in the sham group. In addition, greater motor recovery in the active group might be associated with bigger improvements in measures in activity and participation in the active than in the sham group. Effects of ctDCS_UH_ on cognition or sleep in stroke are largely unknown [23–25]. For this reason, we also assessed, for the first time, measures of cognition and sleep before and after treatment.

Here, we report the results of changes in the following secondary outcome measures of this pilot clinical trial: motor performance, spasticity, and use of the paretic upper limb in activities of daily living, as well as neurological impairment, disability, quality of life, sleep, and cognition.

## 2. Materials and Methods

### 2.1. Design

The study was a randomized parallel, two-arm, double-blind, sham-controlled clinical trial performed at the Albert Einstein Hospital from April, 2015, to September, 2017. The protocol was approved by the hospital's Ethics Committee and registered at clinicaltrials.gov (NCT 024555427). The research was conducted according to standards of the declaration of Helsinki and Brazilian regulations and with institutional guidelines. Informed consent was required from all participants and could be provided in writing by proxies for those unable to sign due to severe motor impairment. The independent Hospital Israelita Albert Einstein Institutional Review Board reviewed the clinical research and informed consent forms, every six months.

### 2.2. Participants

We included subjects in the acute (up to 7 days) or early subacute (from 7 days to 3 months) phases after stroke [19]. Inclusion criteria are as follows: age ≥ 18 years; ischemic stroke at least 72 hours and up to six weeks before enrollment, confirmed by CT or MRI; upper limb paresis defined as a minimum score of 1 in subitem 5a or 5b of the National Institutes of Health Stroke Scale (NIHSS) [26]; and ability to understand the protocol and provide informed consent.

Exclusion criteria are as follows: advanced systemic disease; clinical instability such as uncontrolled cardiac arrhythmia or heart failure; dementia; history of prior stroke affecting the corticospinal tract of M1_UH_; strokes affecting the cerebellum or cerebellar pathways; contraindications to tDCS [27]; Modified Rankin Scale > 2 prior to stroke [26]; pregnancy; contraindication for physical therapy; and comprehension aphasia.

Demographic characteristics, history of hypertension, diabetes mellitus or prior stroke, handedness, performance of thrombolysis for ischemic strokes, time from stroke, and side, type, and etiology of stroke were registered in all subjects. Involvement of primary motor cortex and/or the posterior limb of the internal capsule in brain MRIs (fluid-attenuated inversion recovery images) performed on 3T scanners prior to treatment was also assessed by an experienced neuroradiologist, blinded to group assignment.

### 2.3. Experimental Protocol

#### 2.3.1. Enrolment, Randomization, and Blinding

Recruitment was performed from our hospital admissions and from the community [28]. A computer-generated randomization schedule (10 blocks of 4 subjects) was created with *randomization.com* for allocation to either the active or sham ctDCSM1_UH_ group at a 1 : 1 ratio. Subjects were consecutively enrolled in the study. For instance, if three patients had been included in the study, patient 4 was assigned the condition specified for the fourth included patient. Block randomization assures that a determined proportion of subjects will be included in each group after a certain number of subjects have been included, keeping the proportions of participants in the active and sham groups as similar as possible to desired proportions throughout the study [29].

The randomization table was kept in a locked cabinet and in password-protected files, accessible only to the investigator who administered tDCS and the principal investigator.

Patients and researchers who administered physical therapy or evaluated outcomes were not aware of group assignment.

#### 2.3.2. Intervention

**Figure 1:**
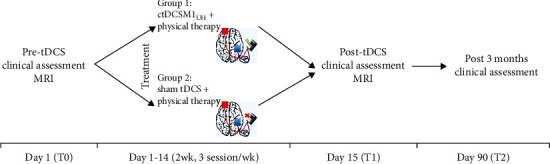
Experimental paradigm. ctDCSM1UH: cathodal transcranial direct stimulation of the motor cortex in the unaffected hemisphere.

Participants underwent three sessions of treatment per week over two weeks (total of six sessions) (Figure 1). In each session, a rubber sponge anode (7 × 5 cm) soaked in saline solution was placed over the ipsilesional supraorbital area and fixed by a nonconducting, nonabsorbent elastic strap. The cathode was placed on the contralesional C3/C4 position according to the EEG 10-20 reference system [27, 30]. The intensity of stimulation was 1 mA, and ramps up and down lasted for 10 seconds (DC-stimulator plus, Neuroconn, Germany).

In the active group, tDCS was applied for 20 minutes, and in the sham group, for 30 seconds including the ramping [30]. The supraorbital region was covered after active or sham ctDCSM1_UH_. This sham setup reduces bias from unblinding [31]. Physical therapy was delivered after the end of stimulation with 30-minute exercises focused on the upper limb (details of the physical therapy interventions are provided as Supplementary Methods and Supplementary Table S1).

To date, there is no consensus or guidelines regarding the optimal intensity (i.e., 1 mA versus 2 mA), interval between sessions (i.e., every other day or consecutive sessions), duration (i.e., 15, 20 min, 30 min, or 40 min), or best timing to deliver physical therapy (concomitant with stimulation versus after the stimulation) [25, 32, 33]. We chose an intensity of 1 mA because higher intensities tend to provoke more paresthesias and could lead to unblinding [3]. We chose a total of 6 sessions with alternate days, in line with the average numbers of sessions (5-10) reported in the literature [12, 14–17]. TDCS was delivered before physical therapy, as performed by other studies that intended to prime cortical excitability prior to motor training [12, 14, 34]. A study about timing of tDCS and robot-aided therapy found that greater effects of tDCS in boosting the effects of training were obtained when tDCS was performed before, compared to during or after training [35].

#### 2.3.3. Outcomes

The primary outcome of this study was safety, and the results were published elsewhere [22]. Secondary efficacy outcomes were assessed before the first session of treatment, after the last session of treatment, and three months later with the following behavioural measures: for upper limb motor impairment, the subitem 5a or 5b of the National Institutes of Health Stroke Scale (NIHSS_5_) [26] and the Upper Limb Fugl-Meyer Assessment of Motor Recovery after Stroke (FMA, maximum motor function score = 66) [36]; for upper limb use in daily living, the Motor Activity Log (MAL) [37]; for upper limb spasticity, the Modified Ashworth Scale (MAS) [38]; for overall neurologic impairment, the National Institutes of Health Stroke Scale total score (NIHSS_total_) [26]; for overall disability, the Modified Rankin Scale (mRS) [26]; for functional independence, the Barthel Index (BI) [26]; for quality of life, the Stroke Impact Scale (SIS) [39]; for cognition, the Montreal Cognitive Assessment (MoCA) [40]; and for sleep, the Pittsburgh Sleep Quality Index (PSQI) questionnaire [41]. Details of the secondary outcomes are provided in the Supplementary Material (Protocol Section: Clinical Outcomes).

#### 2.3.4. Sample Size

Sample size was not formally determined based on prior data because the main goal of this study was to assess safety. Measures of efficacy were secondary outcomes. The results of this pilot study were expected to contribute to sample size estimation for future, larger trials. It has been estimated that, for a parallel, pilot clinical trial, at least 12 subjects should be included per group [42].

#### 2.3.5. Statistical Analysis

Between-group differences in baseline characteristics were assessed with chi-square tests for categorical variables, and unpaired *t*-tests or Mann–Whitney tests for continuous variables according to data distribution.

Outcomes were analyzed with Generalized Estimating Equations (GEE) with factors time (preintervention, postintervention, and after 3 months) and group (active or sham). GEE is used to analyze correlated data, particularly when analysis of variance assumptions are not met [43]. Regarding this model, we used a marginal normal distribution and identity or logarithmic link function for continuous variables [44]. We assumed a Poisson distribution with an identity link function and a first-order autoregressive correlation matrix for discrete variables. Post hoc analyses were performed with Bonferroni's correction for multiple comparisons.

In addition, we evaluated Minimal Clinically Important Differences (MCID) of the following outcomes described for subjects in the early phase post stroke: FMA (9 points) [45], qualitative MAL (1 point) [46], NIHSS (3 points) [47], mRS (1 point) [48], and BI (20 points) [49].

Intention-to-treat (ITT) and per-protocol analyses were performed. Missing observations were imputed with the Last Observation Carried Forward (LOCF). A per-protocol analysis was performed on data from patients who completed at least five sessions of treatment and all sessions of evaluation of outcomes.

## 3. Results

### 3.1. Subjects

Supplementary Figure S1 shows the flow of subjects through the protocol. One subject in the active and one in the sham group dropped out before the assessment of outcomes at baseline. Two subjects in the active and one in the sham group dropped out before the first session of treatment. Eleven subjects completed the treatment in the active and 13 in the sham group. A total of 30 subjects were randomized to either active ctDCSM1UH (*n* = 15) or sham (*n* = 15). There were no significant between-group differences regarding the amount of out-of-protocol physical therapy, during the intervention period or between the end of treatment and the 3-month follow-up (Supplementary Table S1). Information regarding amount of out-of-protocol physical therapy during the intervention period was not available for patients who dropped out. This occurred in only four patients in the active group and 2 patients in the sham group, out of 30 patients included in this study. Considering the period between the end of treatment and the 3-month follow-up, this information was not available for only 2 patients in the active group and 2 patients in the sham group out of 24 patients (Figure S1). Also, there were no between-group differences regarding the number of intervention sessions (*p* = 0.355). None of the subjects dropped out due to adverse events (for details, please see [22]).

Table 1 shows the subjects' characteristics. Lesions affecting the posterior limb of the internal capsule were significantly more frequent in the sham than in the active group. There were no significant differences between the groups at baseline, except for higher FMA scores in the active than in the sham group (*p* < 0.001) according to the GEE model in ITT and per-protocol analyses (Supplementary Tables S2-S4).

### 3.2. Outcomes: Pretreatment, Posttreatment, and Three Months Later

Tables 2 and 3 show the main *ITT* analyses of outcomes, except for SIS and PSQI scores (Supplementary Table S5). In ITT analysis, there were significant GROUP∗TIME interactions, reflecting the overall stronger gains in the sham group for scores in NIHSS_total_, NIHSS_5_, FMA, BI, MoCA, and four SIS domains (“activities of daily living,” “hand function,” “recovery,” and “physical”). Interactions were not statistically significant for mRS, MAS, MAL, and PSQI scores or other SIS domains.

Immediately post treatment, both groups significantly improved compared to pretreatment in NIHSS_total_, NIHSS_5_, FMA, and BI scores, as well as in three SIS domains (“activities of daily living,” “hand function,” and “physical”). MoCA scores improved significantly in the sham group (*p* < 0.001) but not in the active group (*p* > 0.99). The active group improved significantly in the “recovery” SIS domain while no significant change was observed in the sham group.

**Figure 2:**
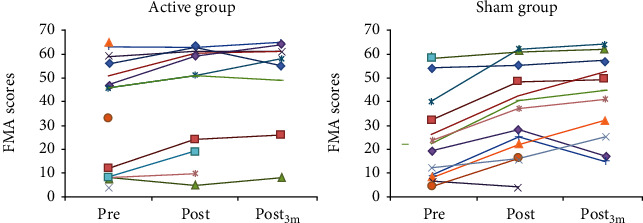
Absolute values of the Fugl-Meyer Assessment (FMA) of motor recovery after stroke scores at specific time points, for each participant in the active and sham groups.

**Figure 3:**
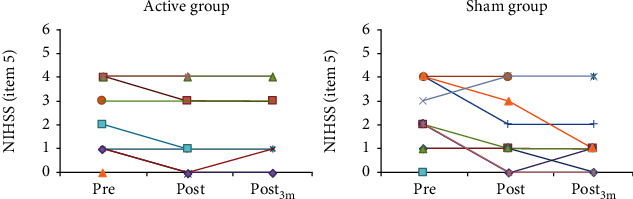
Absolute values of NIHSS (item 5a) scores at specific time points, for each participant in the Active and sham groups. NIHSS: National Institute of Health Stroke Scale.

At three months post intervention, the sham group improved significantly compared to posttreatment in FMA (Figure 2 and Supplementary Figure S2), NIHSS_total_, BI, and three SIS domains (“activities of daily living,” “physical,” and “recovery) while no significant changes occurred in the active group. Also at three months, NIHSS_5_ improved significantly in the sham group and worsened significantly in the active group (Figure 3 and Supplementary Figure S2). NIHSS_5_ scores worsened from 0 pretreatment to 1 posttreatment in two subjects in the active, and in one subject in the sham group. Scores did not change in any other subject in the active and improved in two subjects in the sham group.

Supplementary Tables S3 and S4 show the main *per-protocol* analyses of outcomes, except for SIS and PSQI scores (Supplementary Tables S7-8). The per-protocol analysis showed similar results to ITT, except that there was no significant GROUP∗TIME interaction for NIHSS_total_ and MoCA; at three months post treatment, both groups improved significantly in BI; also at three months, there were no significant changes in NIHSS_5_ in either group.

Due to the imbalance in FMA scores (active > sham at baseline), we performed an additional GEE analysis of this outcome using the baseline FMA score as a covariate. The GROUP∗TIME interaction was no longer significant according to ITT and per-protocol analyses (Supplementary Tables S9-10). Therefore, according to ITT and per-protocol adjusted analyses, both sham and active groups improved significantly at 3 months compared to post treatment.

Table 3 shows MCID results according to the ITT analysis and Supplementary Table S11, to the per-protocol analysis. There were no significant GROUP∗TIME interactions for FMA, NIHSS_total_, BI, MRS, or MAL according to either analysis.

## 4. Discussion

Overall, ctDCSM1_UH_ was not beneficial, compared to sham, in any of the outcomes assessed in this study. There were no significant between-group differences in MCID for FMA, MAL, NIHSS, MRS, or BI. Also, there were no consistent between-group differences in spasticity, use of the paretic limb in activities of daily living, overall neurological impairments, cognition, or quality of sleep. Lower FMA scores in the sham group at baseline were consistent with a greater involvement of the PLIC in this group, compared to the active group. Between-group differences in FMA after treatment favoured the sham group but were no longer statistically significant after adjustments for baseline scores.

The only outcome that improved significantly in the active but not in the sham group was the “recovery” domain of the SIS, according to both ITT and per-protocol analyses. The reason for this finding is unclear, given that no between-group differences were found in other SIS domains or in other outcomes that impact recovery.

On the other hand, performance in the MoCA test improved in the sham but not in the active group, immediately after the end of treatment, according to ITT analysis. The lack of improvement in the active group might reflect a negative effect of ctDCSM1_UH_ on cognition, possibly by disturbing functional connectivity among brain areas other than M1[50, 51], though this speculation remains to be confirmed with imaging and neurophysiological studies. The MoCA test is a screening tool [52], and more comprehensive cognitive evaluations should be included in future protocols of ctDCS in stroke, considering the large knowledge gap in the field.

In opposition to the lack of consistent between-group differences immediately post treatment, at three months later, both ITT and per-protocol analyses showed greater improvements in the sham than in the active group in NIHSS_total_, NIHSS_5_, BI, and three SIS domains (“activities of daily living,” “physical,” and “recovery”). The “physical status” domain evaluates the strength, activity of daily life, mobility, and upper extremity performance. There were no significant between-group differences in any of these outcomes prior to treatment; therefore, these results could point to a detrimental effect of ctDCSM1_UH_. However, the lack of significant differences in MCID for NIHSS_total_ and BI [47] indicates that the better results obtained in the sham than in the active group, at 3 months post treatment, were not clinically relevant.

The number of individuals included per group in this study was greater than in other studies that included patients in the subacute phase, except for Hesse et al. that included 32 patients in each group [13]. Hesse et al. only included patients with severe motor impairments. In contrast, we included subjects with various levels of upper limb involvement. In addition, the chosen experimental paradigm (6 sessions delivered before physical therapy combined with ctDCSM1UH 1 mA intensity and estimated current density of 0.029 mA/cm^2^) had not been previously reported in the subacute phase after stroke.

Despite these differences in the study design compared to prior research in the early phase after stroke, our results point to the same direction of all studies that chose stimulus intensities *below* 2 mA and estimated current densities *below* 0.057 mA/cm^2^: we did not find significant between-group differences in outcomes related to upper limb impairment, function, overall neurologic impairment, or disability, immediately after the end of treatment. These results are consistent with a meta-analysis with substantial heterogeneity (*I*^2^ = 63.8) [8], indicating that ctDCSM1_UH_ does not lead to long-term enhancement of upper limb function when delivered at an early stage post stroke.

Plasticity mechanisms are highly active during the first weeks after stroke. It is possible that ctDCSM1_UH_ during this critical period does not have a positive impact on these mechanisms and the interhemispheric inhibition theory does not play an important role in many patients with stroke as previously argued [53–57]. Instead of being maladaptive, the activity of M1_UH_ may be relevant to motor performance and recovery in these subjects, and hence, administration of ctDCSM1_UH_ may be ineffective. The magnitude of endogenous plasticity may be higher than any effects of ctDCSM1_UH_ during the first weeks and months.

On the other hand, Khedr et al. [14] (2 mA for 25 min, 6 sessions) found marginally positive results after treatment with ctDCS compared to sham in outcomes related to upper limb impairment. This could be related to paradigm choice: the timing of stimulation was performed in an earlier time window (17 days) than (around 4 weeks), and the current intensity was 2 mA. Only subjects who presented motor-evoked potentials in a hand muscle were included. Another study [15] also applied a 2 mA current intensity but reported positive results only after 6 months of follow-up in subjects with mild motor impairments. Estimated current densities were, respectively, 0.057 and 0.08 mA/cm^2^. Conversely, Hesse et al. [13] chose a stimulus intensity of 2 mA (current density, 0.057 mA/cm^2^) and did not report significant differences between active and sham groups but only included subjects with severe motor deficits.

The lack of significant effects of cTDCS in the early phase after stroke [58] may reflect the mechanisms of recovery, but it is also possible that paradigms of stimulation and eligibility criteria may explain discrepancies in results. A metaregression estimated that, overall, higher tDCS current densities may lead to greater motor recovery [59]. In addition, in patients in the chronic phase after stroke, an intensity of 4 mA was found to be safe [60], but until now, no studies evaluated intensities greater than 2 mA in earlier stages.

Overall, these results, together with our observations, provide key information for the design of future studies aiming at efficacy on motor outcomes: administration of ctDCSM1_UH_ at stimulus intensities of at least 2 mA, current densities greater than 0.057 mA/cm^2^ in patients with residual upper function, without severe deficits, may be associated with a greater likelihood of success. Tailoring the type of tDCS (cathodal or anodal) to each individual according to the severity of their deficits and/or phase after stroke is more likely to lead to benefit than applying these treatments to very different groups of subjects with stroke, a very heterogeneous condition. Clinical, neuroimaging, and neurophysiologic tools are expected to provide information about the underlying mechanisms of recovery that will allow the selection of the right patient to the right intervention at the right dose [61].

These conclusions cannot be extrapolated to other neuromodulation interventions such as rTMS [62, 63] or anodal tDCS administered during the first days and weeks after stroke. For instance, Andrade et al. [11] reported that functional independence assessed with the Barthel Index improved significantly more after anodal tDCS of the premotor or primary motor cortex in the affected hemisphere than after sham stimulation, in subjects at 1-3 months after stroke. In the three groups, tDCS was followed by constraint-induced movement therapy (CIMT) in 10 sessions of treatment. Subjects were recruited at a later subacute stage compared to those included in the present study. Differences in treatment schedule, time after stroke, type and target of tDCS, outcome (functional independence), and add-on rehabilitation paradigm (CIMT) may also explain discrepancies between these results and the findings of the present study.

These results should be viewed with caution considering the limitations of this study. First, behavioral measures were secondary outcomes and were collected in a relatively small sample of patients with a main goal of assessing the estimate of effect that would allow a formal sample size calculation for a further larger study. Second, we did not conduct a stratified randomization according to the level of impairment, and there was an imbalance in FMA scores at baseline. However, we also analyzed the data considering baseline FMA as a covariate, and there were no between-group differences. Third, biomarkers such as cortical excitability or severity of motor impairment were not part of the eligibility criteria. Until now, there is no consensus about evidence-based biomarkers that should be used in trials of neuromodulation in stroke. There is a deep need for clinical, imaging, or other variables that can help tailor treatments [64]. Another potential limitation is that the principal investigator, and not an independent investigator, sent the information about the computer-generated randomization schedule to the researcher who administered tDCS. Finally, the duration of upper limb therapy may have been insufficient. There is still no consensus about the best duration of upper limb therapy combined with tDCS in the subacute stage after stroke. The VECTORS study showed an absence of benefit of an intensive intervention of motor training, constraint-induced movement therapy, compared to usual care in the subacute phase after stroke [65], in the absence of add-on neuromodulation interventions. This finding contrasts with results of CIMT in patients in the late subacute and chronic stages after stroke [66]. Yet, it is possible that tDCS may be beneficial when longer durations of training are provided, compared to those administered in our study. It is also possible that fatigue in subacute patients or maladaptive effects of “excessive” stimulation with prolonged training limit the potential benefits of longer therapy sessions. Future studies are necessary to define not only the dose of CtDCSM1_UH_ but also the optimal “dose” of therapy applied in combination with CtDCSM1_UH_.

## 5. Conclusion

In summary, our data provide evidence that CtDCSM1_UH_ in the early phase after stroke did not have consistent beneficial effects on motor impairments, disability, or quality of life, immediately after treatment or three months later. Early phase dose-finding studies after stroke are necessary before planning larger clinical trials.

## Figures and Tables

**Table 1:** Characteristics of the subjects.

Characteristic	Active tDCS (*n* = 15)	Sham tDCS (*n* = 15)	*p* value
Gender (female/male)	8/7	4/11	0.136^1^
Age, years (mean ± SD)	61.8 ± 15	61.9 ± 17.9	0.991^2^
Education, years (mean ± SD)	9.3 ± 4.1	7.5 ± 4.9	0.305^3^
Ethnicity, *n* (%)			0.478^4^
White	9 (60)	9 (60)	
Black	6 (40)	5 (33.3)	
Asian	0 (0)	1 (6.7)	
Hypertension, *n* (%)	10 (66.7)	12 (80)	0.682^5^
Diabetes mellitus, *n* (%)	7 (46.7)	6 (40)	0.713^1^
Right-handedness, *n* (%)	12 (85.7)	13 (92.9)	>0.999^5^
Previous stroke, *n* (%)	2 (13.3)	1 (7.1)	>0.999^5^
Time since stroke, median (IQR)	37 (23.5; 45.5)	26.5 (20.8; 37.3)	0.155^3^
Thrombolysis, *n* (%)	3 (20)	2 (13.3)	>0.999^5^
Lesion side (right/left/bilateral)	7; 8; 0	7; 7; 1	0.484^4^
HADS—depression, median (IQR)	3 (1; 6.5)	1.5 (0; 5.3)	0.246^3^
HADS—total score, median (IQR)	9 (4; 12)	4 (2; 11)	0.114^3^
Lesion site			
Corticosubcortical	9 (60)	5 (35.7)	0.191^1^
Subcortical	6 (40)	9 (64.3)	0.191^1^
Involved M1	6 (40)	4 (28.6)	0.700^5^
Involved PLIC	8 (53.3)	13 (92.9)	0.035^5^
Stroke etiology, TOAST			0.610^4^
Large-artery atherosclerosis	2 (13.4)	2 (13.3)	
Small-vessel occlusion	0 (0)	1 (6.7)	
Other determined etiology	2 (13.4)	1 (6.7)	
Undetermined etiology^a^	1 (6.7)	1 (6.7)	
Undetermined etiology^b^	10 (66.7)	10 (66.7)	

tDCS: transcranial direct current stimulation. HADS: Hospital Anxiety Depression Scale. SD: standard deviation. IQR: interquartile range. M1: primary motor cortex. PLIC: posterior limb of the internal capsule. TOAST: according to criteria from the Trial of Org 10172 in Acute Stroke Treatment. ^1^Chi-square test. ^2^Student's *t*-test. ^3^Mann-Whitney's test. ^4^Likelihood ratio test. ^5^Fisher's exact test. ^a^Complete investigation.^.b^Incomplete investigation.

**Table 2:** Outcomes assessed before the first session of treatment (Pre), after the last session of treatment (Post), and three months later (Post_3m_): intention-to-treat analysis, Generalized Estimating Equation model. Median and interquartile ranges are given.

	Active			Sham			*p* values		
Outcomes	Pre	Post	Post_3m_	Pre	Post	Post_3m_	Group	Time	Interaction
NIHSS_total_	6 (3; 13)	3 (3; 11)	4 (3; 11)	5 (4; 10)	5 (3; 10)	4 (1; 8)	0.173	<0.001	<0.001
NIHSS_5_	2 (1; 4)	1 (0; 4)	1 (1; 4)	2 (1; 4)	1 (1; 4)	1 (1; 4)	0.866	<0.001	<0.001
FMA	46 (8; 56.8)	51 (16.8; 61.5)	52 (16.8; 61.8)	22.5 (8.8; 43.5)	38.5 (20.5; 55.8)	43 (16.8; 57.3)	0.015	<0.001	<0.001
mRS	3 (2; 4)	3 (2; 4)	3 (2; 4)	4 (3; 4)	3 (3; 3)	3 (2; 3)	0.689	0.012	0.910
BI	80 (47.5; 95)	85 (57.5; 100)	92.5 (61.3; 100)	65 (47.5; 77.5)	77.5 (67.5; 90)	85 (75; 100)	0.654	<0.001	<0.001
MAS_shoulder_	0 (0; 1)	0 (0; 0)	0 (0; 0)	0 (0; 1)	0 (0; 0)	0 (0; 0.5)	0.717	0.010	0.176
MAS _elbow_	0 (0; 1.25)	0.5 (0; 1)	0.5 (0; 1.25)	1 (0; 2)	1 (0; 2)	1 (0; 2)	0.279	0.588	0.975
MAS _wrist_	0.5 (0; 2.25)	0 (0; 1)	0 (0; 1.25)	1 (0.8; 2)	1 (0; 2)	2 (0; 2)	0.148	0.039	0.296
MAS _fingers_	0.5 (0; 1.25)	0 (0; 1)	0 (0; 1)	1 (0; 1)	0 (0; 1)	1 (0; 1.3)	0.587	0.016	0.702
MAL_quantitative_	1.05 (0; 1.97)	2.41 (0; 3.5)	2.25 (0; 3.89)	0.1 (0; 0.4)	0.6 (0; 1.9)	0.8 (0; 3.5)	0.261	0.087	0.211
MAL_qualitative_	0.89 (0; 1.67)	2.16 (0; 3.58)	2.41 (0; 3.65)	0 (0; 0.2)	0.7 (0; 1.3)	0.9 (0; 3.1)	0.264	0.095	0.183
MoCA	18 (9; 24)	19 (10; 23)	21 (8; 24)	16 (8; 20)	20 (12; 23)	19 (13; 23)	0.728	<0.001	0.001

tDCS: transcranial direct current stimulation. NIHSS _total_: National Institutes of Health Stroke Scale total score (0-42). NIHSS_5_: National Institutes of Health Stroke Scale, motor score (0-5). FMA: Fugl-Meyer Assessment of Motor Recovery after Stroke, upper limb motor score. mRS: Modified Rankin Scale. BI: Barthel Index. MAS: Modified Ashworth Scale. MAL_qualitative_: subscale qualitative of Motor Activity Log. MAL_quantitative_: subscale quantitative of Motor Activity Log. MoCA: Montreal Cognitive Assessment.

**Table 3:** Minimal clinically important differences for secondary outcomes. Intention-to-treat analysis, Generalized Estimating Equations model with binomial distribution.

Outcome	Active		Sham		*p* value		
	Pre-post	Post-3m	Pre-post	Post-3m	Group time interaction		
	*n* (%)	*n* (%)	*n* (%)	*n* (%)			
Fugl-Meyer Assessment	6 (42.9)	0 (0)	8 (57.1)	0 (0)	0.727	0.001	0.727
Motor activity log, qualitative	5 (35.7)	0 (0)	4 (28.6)	3 (21.4)	0.433	0.068	0.114
National Institutes of Health Stroke Scale	2 (13.3)	0 (0)	2 (13.3)	2 (13.3)	0.421	0.839	0.472
Modified Rankin Scale	5 (33.3)	4 (26.7)	7 (46.7)	4 (26.7)	0.576	0.328	0.647
Barthel Index	1 (7.1)	0 (0)	2 (14.3)	1 (7.1)	0.382	0.967	0.987

## Data Availability

The data that support the findings of this study are available from the corresponding author upon request.

## Supplementary Materials

Supplementary Materials Figure S1. Flow of subjects throughout the study, Figure S2. Changes in Fugl-Meyer Assessment of Motor Recovery after Stroke (FMA) and NIH Stroke Scale (item 5) scores at specific time points, according to intention to treat (ITT, left) and per protocol (right) analyses. Error bars represent the standard error for 15 patients in each group (n= 30, ITT, left), 9 patients in the active group and 11 patients in the sham group (n=20, per protocol, right). ∗ Statistically significant differences. Table S1. Hours of physical therapy out of protocol as reported by the patients, between the first and last session of transcranial direct current stimulation (tDCS) and between the last session and three months later. Table S2. Post-hoc intention-to-treat analyses of: motor subitem (5a) of the National Institutes of Health Stroke Scale, Fugl-Meyer Assessment of Motor Recovery after Stroke -Upper limb motor score, Modified Ashworth Scale, Barthel Index, National Institutes of Health Stroke Score (total scale) and Modified Rankin Scale. Table S3. Outcomes assessed before the first session of treatment (Pre), after the last session of treatment (Post) and three months later (Post_3m_): per-protocol analysis, Generalized Estimating Equation model. Median and interquartile ranges are given. Table S4. Post-hoc per-protocol analyses of: Motor subitem (5a) of the National Institutes of Health Stroke Scale, Fugl-Meyer Assessment of Motor Recovery after Stroke - Upper limb motor score, Modified Ashworth Scale, Barthel Index, Motor Activity Log. Table S5. Stroke Impact Scale (SIS) and Pittsburgh Sleep Quality Index assessed before the first session of treatment (Pre), after the last session of treatment (Post) and three months later (Post_3m_): intent-to-treat analysis, Generalized Estimating Equation model. Median and interquartile ranges are given. Table S6. Stroke Impact Scale (SIS). Post-hoc, intention-to-treat analysis. Table S7. Stroke Impact Scale (SIS) and Pittsburgh Sleep Quality Index assessed before the first session of treatment (Pre), after the last session of treatment (Post) and three months later (Post_3m_): per-protocol analysis, Generalized Estimating Equation model. Median and interquartile ranges are given. Table S8. Stroke Impact Scale (SIS). Post-hoc, per-protocol analysis. Table S9. Generalized Estimating Equations, intention-to-treat (ITT) and per-protocol analyses of Fugl-Meyer Assessment (FMA) scores, adjusted for pre-treatment FMA scores (covariate). Table S10. Post-hoc intention-to-treat and per-protocol analyses of Fugl-Meyer Assessment (FMA) scores, adjusted for baseline FMA scores (covariate). Table S11. Minimal clinically important difference. Per-protocol analysis of changes before and after treatment (Pre-Post), as well as post-treatment compared to 3 months after treatment (Post-Post_3m_).
