# Supplementary material for: Contralesional Cathodal Transcranial Direct Current Stimulation Does Not Enhance Upper Limb Function in Subacute Stroke: A Pilot Randomized Clinical Trial
Source: Neural Plast. 2021 Aug 10;2021:8858394. doi: 10.1155/2021/8858394 (PMC8380180; doi:10.1155/2021/8858394)
Supplement: Supplementary Materials — Figure S1. Flow of subjects throughout the study, Figure S2. Changes in Fugl-Meyer Assessment of Motor Recovery after Stroke (FMA) and NIH Stroke Scale (item 5) scores at specific time points, according to intention to treat (ITT, left) and per protocol (right) analyses. Error bars represent the standard error for 15 patients in each group (n= 30, ITT, left), 9 patients in the active group and 11 patients in the sham group (n=20, per protocol, right). ∗ Statistically significant differences. Table S1. Hours of physical therapy out of protocol as reported by the patients, between the first and last session of transcranial direct current stimulation (tDCS) and between the last session and three months later. Table S2. Post-hoc intention-to-treat analyses of: motor subitem (5a) of the National Institutes of Health Stroke Scale, Fugl-Meyer Assessment of Motor Recovery after Stroke -Upper limb motor score, Modified Ashworth Scale, Barthel Index, National Institutes of Health Stroke Score (total scale) and Modified Rankin Scale. Table S3. Outcomes assessed before the first session of treatment (Pre), after the last session of treatment (Post) and three months later (Post3m): per-protocol analysis, Generalized Estimating Equation model. Median and interquartile ranges are given. Table S4. Post-hoc per-protocol analyses of: Motor subitem (5a) of the National Institutes of Health Stroke Scale, Fugl-Meyer Assessment of Motor Recovery after Stroke - Upper limb motor score, Modified Ashworth Scale, Barthel Index, Motor Activity Log. Table S5. Stroke Impact Scale (SIS) and Pittsburgh Sleep Quality Index assessed before the first session of treatment (Pre), after the last session of treatment (Post) and three months later (Post3m): intent-to-treat analysis, Generalized Estimating Equation model. Median and interquartile ranges are given. Table S6. Stroke Impact Scale (SIS). Post-hoc, intention-to-treat analysis. Table S7. Stroke Impact Scale (SIS) and Pittsburgh Sleep Quality Ind [file 8858394.f1.docx]

**SUPPLEMENTARY MATERIAL**

| **Supplementary Figures** | **Pages 2-3** |
| --- | --- |
| **Supplementary Methods** | **Page 4** |
| **Supplementary Tables** | **Pages 5-20** |
| **Protocol** | **Pages 21-37** |

**Supplementary Figures**

Figure S1. Flow of subjects throughout the study

**Inclusion Criteria**

**No**

**(n=125)**

**Assessed for eligibility**

**(n=187)**

**Yes**

**(n=62)**

**Yes**

**(n=32)**

**Exclusion Criteria**

**Randomization**

**(n=30)**

**Active tDCS**

**(n=15)**

**Sham tDCS**

**(n=15)**

**Allocation**

**Follow-up**

**4 drop- outs**

-1 patient with uncontrolled hypertension

-1 patient with deep venous thrombosis

-2 patients for personal reasons

**2 drop- outs**

- 1 patient with urinary tract infection/sepsis

-1 patient for abdominal pain

**Completed Intervention**

**(n=11)**

**Completed Intervention**

**(n=13)**

**Post-intervention**

**(n=11)**

**Post-intervention**

**(n=13)**

**3-month Assessment**

**(n=9)**

**3-month Assessment**

**(n=11)**

**Lost to follow-up**

**(n=2)**

**Lost to follow-up**

**(n=2)**

Figure S2. Changes in Fugl-Meyer Assessment of Motor Recovery after Stroke (FMA) and NIH Stroke Scale (item 5) scores at specific time points, according to intention to treat (ITT, left) and per protocol (right) analyses. Error bars represent the standard error for 15 patients in each group (n= 30, ITT, left), 9 patients in the active group and 11 patients in the sham group (n=20, per protocol, right). * Statistically significant differences.

*

**Supplementary Methods**

A blinded therapist administered, for 50 minutes, exercises selected from a standard program and adapted to individual needs. The activities are listed below.

Lower limbs

-Sensory stimulation: in the supine position, physical therapist slides a towel over the affected limb.

-Passive mobilization of lower limbs.

In the supine position, patient performs triple flexion abduction and hip adduction, dorsiflexion and bilateral ankle plantar flexion (3 repetitions).

-Stretching: Hamstrings, sural triceps, hip flexor, adductors.

-Strengthening: Hip extensors, foot dorsiflexors, knee flexors and abdominal muscles.

-Functional training: Sitting-to-standing.

Upper limbs

-Sensory stimulation: in the supine position, physical therapist slides a towel over the affected limb.

-Passive mobilization of upper limbs.

-Stretching: Shoulder flexors and adductors, elbow flexors, wrist flexors, wrist extensors, shoulder extensors and elbow extensors.

-Functional training: Anterior and lateral reach exercises.

We registered the amount of out-of-protocol physical therapy, during treatment and for the next three months after the end of treatment.

**Supplementary Tables**

Table S1. Hours of physical therapy out of protocol as reported by the patients, between the first and last session of transcranial direct current stimulation (tDCS) and between the last session and three months later

| Physical therapy out of protocol | Active | Sham | P-value |
| --- | --- | --- | --- |
| Between the first and last session of tDCS |  |  |  |
| Number of patients, n (%) | 5 (45.5) | 5 (38.5) | 0.729^1^ |
| Hours of physical therapy (median and range) | 3 (1.0; 3.0) | 2 (0.7; 2.5) | 0.222^2^ |
| Between the last session of tDCS and three months later |  |  |  |
| Number of patients, n (%) | 5 (55.6) | 9 (81.8) | 0.201^1^ |
| Number of sessions of physical therapy (median and range) | 24 (20; 60) | 24 (4; 72) | 0.797^2^ |

^1^Chi-square test. ^2^Mann-Whitney test.

^#^No information available for 4 patients in the active group and 2 patients in the sham group between the first and last session of tDCS, and for 6 patients in the active group and 4 patients in the sham group between the last session of tDCS and three months later. Two subjects in the sham group and one in the active group underwent occupational therapy; another one in the active group underwent constraint-induced movement therapy.

**Table S2. Post-hoc intention-to-treat analyses of: motor subitem (5a) of the National Institutes of Health Stroke Scale, Fugl-Meyer Assessment of Motor Recovery after Stroke -Upper limb motor score, Modified Ashworth Scale, Barthel Index, National Institutes of Health Stroke Score (total scale) and Modified Rankin Scale.**

| Group/Moment | Comparison^£^ | | Mean difference | Standard error | P-value | CI (95%) | |  |  |  |  |
| --- | --- | --- | --- | --- | --- | --- | --- | --- | --- | --- | --- |
|  |  |  |  |  |  | Inferior | Superior |  |  |  |  |
| Motor subitem (5a) of the National Institutes of Health Stroke Scale | | | | | | | |  |  |  |  |
| Active | Post | Pre | -0.40 | 0.05 | <0.001 | -0.56 | -0.24 |  | |  |  |
|  | Post_3m_ | Post | 0.13 | 0.04 | 0.018 | 0.01 | 0.25 |  | |  |  |
| Sham | Post | Pre | -0.53 | 0.06 | <0.001 | -0.72 | -0.35 |  | |  |  |
|  | Post_3m_ | Post | -0.13 | 0.04 | 0.018 | -0.25 | -0.01 |  | |  |  |
| Pre | Active | Sham | -0.27 | 0.57 | >0.999 | -1.93 | 1.39 |  |  |  |  |
| Post | Active | Sham | -0.13 | 0.51 | >0.999 | -1.62 | 1.36 |  |  |  |  |
| Post_3m_ | Active | Sham | 0.13 | 0.51 | >0.999 | -1.36 | 1.62 |  |  |  |  |
| Fugl-Meyer Assessment of Motor Recovery After Stroke, Upper limb motor score | | | | | | | |  |  |  |  |
| Active | Post | Pre | 4.43 | 0.38 | <0.001 | 3.31 | 5.55 |  | |  | |
|  | Post_3m_ | Post | 0.71 | 0.38 | 0.934 | -0.41 | 1.84 |  | |  | |
| Sham | Post | Pre | 10.21 | 0.41 | <0.001 | 9 | 11.43 |  | |  | |
|  | Post_3m_ | Post | 1.64 | 0.37 | <0.001 | 0.56 | 2.72 |  | |  | |
| Pre | Active | Sham | 9.64 | 2.12 | <0.001 | 3.43 | 15.85 |  |  |  |  |
| Post | Active | Sham | 3.86 | 2.35 | >0.999 | -3.04 | 10.75 |  |  |  |  |
| Post_3m_ | Active | Sham | 2.93 | 2.39 | >0.999 | -4.07 | 9.93 |  |  |  |  |
| Modified Ashworth Scale (shoulder) | | | | | | | |  |  |  |  |
| Active/Sham | Post | Pre | -0.25 | 0.1 | 0.042 | -0.49 | -0.01 |  | |  | |
|  | Post_3m_ | Post | 0.18 | 0.1 | 0.198 | -0.05 | 0.41 |  | |  | |
| Modified Ashworth Scale (wrist) | | | | | | | |  |  |  |  |
| Active/Sham | Post | Pre | -0.36 | 0.17 | 0.121 | -0.77 | 0.06 |  |  |  |  |
|  | Post_3m_ | Post | 0.29 | 0.17 | 0.274 | -0.12 | 0.69 |  |  |  |  |
| Modified Ashworth Scale (fingers) | | | | | | | |  |  |  |  |
| Active/Sham | Post | Pre | -0.21 | 0.12 | 0.222 | -0.5 | 0.07 |  |  |  |  |
|  | Post_3m_ | Post | 0.29 | 0.12 | 0.065 | -0.01 | 0.58 |  |  |  |  |
| Barthel Index | | | | | | | |  |  |  |  |
| Active | Post | Pre | 8.21 | 0.77 | <0.001 | 5.96 | 10.47 |  |  |  |  |
|  | Post_3m_ | Post | 1.79 | 0.79 | 0.343 | -0.52 | 4.09 |  |  |  |  |
| Sham | Post | Pre | 14.29 | 0.77 | <0.001 | 12.02 | 16.55 |  |  |  |  |
|  | Post_3m_ | Post | 6.79 | 0.8 | <0.001 | 4.44 | 9.13 |  |  |  |  |
| Pre | Active | Sham | 7.14 | 3.06 | 0.296 | -1.85 | 16.14 |  |  |  |  |
| Post | Active | Sham | 1.07 | 3.32 | >0.999 | -8.66 | 10.8 |  |  |  |  |
| Post_3m_ | Active | Sham | -3.93 | 3.41 | >0.999 | -13.93 | 6.07 |  |  |  |  |
| NIH Stroke Scale, total score | | | | | | | |  |  |  |  |
| Active | Post | Pre | -1.07 | 0.1 | <0.001 | -1.37 | -0.76 |  |  |  |  |
|  | Post_3m_ | Post | -0.13 | 0.09 | >0.999 | -0.39 | 0.12 |  |  |  |  |
| Sham | Post | Pre | -0.93 | 0.1 | <0.001 | -1.22 | -0.65 |  |  |  |  |
|  | Post_3m_ | Post | -0.67 | 0.09 | <0.001 | -0.92 | -0.41 |  |  |  |  |
| Pre | Active | Sham | 1.13 | 0.96 | >0.999 | -1.68 | 3.95 |  |  |  |  |
| Post | Active | Sham | 1 | 0.89 | >0.999 | -1.6 | 3.6 |  |  |  |  |
| Post_3m_ | Active | Sham | 1.53 | 0.86 | >0.999 | -0.98 | 4.05 |  |  |  |  |
| Modified Rankin Scale | | | | | | | |  |  |  |  |
| Active/Sham | Post | Pre | -0.4 | 0.15 | 0.028 | -0.77 | -0.03 |  |  |  |  |
|  | Post_3m_ | Post | -0.2 | 0.15 | 0.508 | -0.55 | 0.15 |  |  |  |  |

CI: confidence interval. £:Comparison is performed between two different time intervals: after treatment compared to before treatment (Post- Pre) and 3 months after treatment compared to post-treatment (Post_3m_ – Post) for each group. We also compared active minus sham values at pre, post and post_3m_ moments for different scales.

Table S3. Outcomes assessed before the first session of treatment (Pre), after the last session of treatment (Post) and three months later (Post_3m_): per-protocol analysis, Generalized Estimating Equation model. Median and interquartile ranges are given

| Outcome | Active | | | Sham | | | P-value | | | | |
| --- | --- | --- | --- | --- | --- | --- | --- | --- | --- | --- | --- |
|  | Pre | Post | Post_3m_ | Pre | Post | Post_3m_ | Group | Time | Interaction | | |
| NIHSS_total_ | 4 (3; 8) | 3 (3; 6) | 3 (2.5; 6) | 5 (4; 6) | 4 (3; 5) | 3 (1; 5) | 0.799 | <0.001 | | 0.117 |  |
| NIHSS_5_ | 1 (1; 3.5) | 1 (0; 3) | 1 (0.5; 3) | 2 (1; 4) | 1 (1; 3) | 1 (1; 2) | 0.694 | <0.001 | | 0.013 |  |
| FMA | 47 (29; 57.5) | 59 (37.5; 62) | 58 (37.5; 62.5) | 23 (12; 40) | 40 (25; 55) | 45 (25; 57) | <0.001 | <0.001 | | <0.001 |  |
| mRS | 3 (2; 4) | 2 (2; 3.5) | 2 (1; 3) | 3 (3; 4) | 3 (3; 3) | 3 (2; 3) | 0.556 | 0.027 | | 0.967 |  |
| BI | 90 (50; 95) | 95 (70; 100) | 100 (75; 100) | 65 (50; 85) | 85 (75; 90) | 90 (80; 100) | 0.678 | <0.001 | | <0.001 |  |
| MAS_shoulder_ | 0 (0; 0.5) | 0 (0; 0) | 0 (0; 0) | 0 (0; 1) | 0 (0; 0) | 0 (0; 0) | 0.163 | 0.331 | | 0.550 |  |
| MAS _elbow_ | 0 (0; 2.5) | 0 (0; 1) | 0 (0; 1) | 1 (1; 2) | 1 (0; 2) | 1 (0; 2) | 0.107 | 0.195 | | 0.652 |  |
| MAS _wrist_ | 1 (0; 3) | 0 (0; 1) | 0 (0; 1.5) | 1 (1; 2) | 1 (0; 2) | 2 (0; 2) | 0.381 | 0.020 | | 0.210 |  |
| MAS _fingers_ | 1 (0; 1.5) | 0 (0; 1) | 0 (0; 1.5) | 1 (0; 1) | 0 (0; 1) | 1 (0; 1) | 0.661 | 0.016 | | 0.922 |  |
| MAL_quantitative_ | 1.31(0.42; 1.98) | 3.05(0.99; 3.63) | 2.91 (1.11; 3.99) | 0.2 (0; 0.3) | 0.7 (0.1; 2.2) | 1 (0; 3.7) | <0.001 | <0.001 | | 0.279 |  |
| MAL_qualitative_ | 1.4(0.3; 1.74) | 3.12 (0.67; 3.65) | 2.75 (1.14; 3.74) | 0.1(0; 0.1) | 0.7 (0.3; 1.6) | 1 (0.1; 3.1) | 0.219 | 0.056 | | 0.130 |  |
| MoCA | 21 (11.5; 24,5) | 21 (16.5; 26) | 23 (15; 25.5) | 17 (13.3; 20,8) | 20.5(12.3; 25.3) | 20.5(14.8; 23.8) | 1.386 | <0. 002 | | 1.156 |  |

NIHSS _total_: National Institutes of Health Stroke Scale total score (0-42). NIHSS_5_: National Institute of Health Stroke Scale motor score (0-5). mRS: modified Rankin Scale. BI: Barthel Index. MAS: modified Ashworth Scale. MAL _quantitative_: subscale quantitative of Motor Activity Log MAL _qualitative_: subscale qualitative of Motor Activity Log. MoCA: Montreal Cognitive Assessment.

Table S4. Post-hoc per-protocol analyses of: Motor subitem (5a) of the National Institutes of Health Stroke Scale,

Fugl-Meyer Assessment of Motor Recovery after Stroke - Upper limb motor score, Modified Ashworth Scale, Barthel Index, Motor Activity Log.

| Group/Moment | Comparison^£^ | | Mean difference | | | | Standard error | P-value | CI (95%) | |
| --- | --- | --- | --- | --- | --- | --- | --- | --- | --- | --- |
|  |  | |  | | | |  |  | Inferior | Superior |
| Motor subitem (5a) of the National Institutes of Health Stroke Scale | | | | | | | | | | |
| Active | Post | Pre | | | | -0.56 | 0.13 | <0.001 | -0.93 | -0.18 |
|  | Post_3m_ | Post | | | | 0.22 | 0.11 | 0.526 | -0.09 | 0.53 |
| Sham | Post | Pre | | | | -0.73 | 0.13 | <0.001 | -1.11 | -0.34 |
|  | Post_3m_ | Post | | | | -0.18 | 0.10 | 0.911 | -0.47 | 0.1 |
| Pre | Active | Sham | | | | -0.47 | 0.65 | >0.999 | -2.39 | 1.44 |
| Post | Active | Sham | | | | -0.30 | 0.55 | >0.999 | -1.90 | 1.30 |
| Post_3m_ | Active | Sham | | | | 0.10 | 0.55 | >0.999 | -1.52 | 1.72 |
|  |  |  | | | |  |  |  |  |  |
| Fugl-Meyer Assessment, Upper limb | | | | | | | | | | |
| Active | Post | Pre | | | 5.44 | | 0.65 | 0.001 | 3.53 | 7.36 |
|  | Post_3m_ | Post | | | 1.11 | | 0.66 | >0.999 | -0.83 | 3.06 |
| Sham | Post | Pre | | | 12.09 | | 0.58 | <0.001 | 10.38 | 13.81 |
|  | Post_3m_ | Post | | | 2.09 | | 0.55 | 0.002 | 0.48 | 3.7 |
| Pre | Active | Sham | | | 15.57 | | 2.70 | <0.001 | 7.64 | 23.49 |
| Post | Active | Sham | | | 8.92 | | 3.00 | 0.044 | 0.11 | 17.72 |
| Post_3m_ | Active | Sham | | | 7.94 | | 3.05 | 0.139 | -1.02 | 16.90 |
|  |  |  | | |  | |  |  |  |  |
| Modified Ashworth Scale (wrist) | | | | | | | | | | |
| Active/Sham | Post | Pre | -0.54 | | | | 0.23 | 0.063 | -1.09 | 0.02 |
|  | Post_3m_ | Post | 0.38 | | | | 0.22 | 0.226 | -0.13 | 0.9 |
| Modified Ashworth Scale (fingers) | | | | | | | | | | |
| Active/Sham | Post | Pre | | -0.25 | | | 0.15 | 0.301 | -0.61 | 0.11 |
|  | Post_3m_ | Post | | 0.39 | | | 0.16 | 0.046 | 0 | 0.78 |
| Barthel Index |  |  | |  | | |  |  |  |  |
| Active | Post | Pre | | 8.33 | | | 0.79 | <0.001 | 6.02 | 10.64 |
|  | Post_3m_ | Post | | 2.78 | | | 0.80 | 0.008 | 0.43 | 5.13 |
| Sham | Post | Pre | | 15.45 | | | 0.73 | <0.001 | 13.32 | 17.59 |
|  | Post_3m_ | Post | | 8.64 | | | 0.74 | <0.001 | 6.45 | 10.82 |
| Pre | Active | Sham | | 8.33 | | | 3.75 | 0.394 | -2.67 | 19.34 |
| Post | Active | Sham | | 1.21 | | | 4.05 | >0.999 | -10.67 | 13.09 |
| Post_3m_ | Active | Sham | | -4.65 | | | 4.18 | >0.999 | -16.92 | 7.63 |
|  |  |  | |  | | |  |  |  |  |
| Motor Activity Log, quantitative | | | | | | | | | | |
| Pre/Post/Post_3m_ | Active | Sham | | 0.84 | | | 0.24 | 0.001 | 0.37 | 1.32 |
| Active/Sham | Post | Pre | | 0.99 | | | 0.32 | 0.002 | 0.36 | 1.61 |
|  | Post_3m_ | Post | | 0.26 | | | 0.25 | 0.314 | -0.24 | 0.76 |

CI: confidence interval. £: Comparison is performed between two different time intervals: after treatment compared to before treatment (Post- Pre) and 3 months after treatment compared to post-treatment (Post_3m_ – Post) for each group. We also compared active minus sham values at pre, post and post_3m_ moments for different scales.

Table S5. Stroke Impact Scale (SIS) and Pittsburgh Sleep Quality Index assessed before the first session of treatment (Pre), after the last session of treatment (Post) and three months later (Post_3m_): intent-to-treat analysis, Generalized Estimating Equation model. Median and interquartile ranges are given.

| Outcome | Active | | | Sham | | | P-value | | |
| --- | --- | --- | --- | --- | --- | --- | --- | --- | --- |
|  | Pre | Post | Post_3m_ | Pre | Post | Post_3m_ | Group | Time | Interaction |
| SIS Domains | | |  |  |  |  |  |  |  |
| *Strength* | 50 | 50 | 56.3 | 25 | 56.3 | 56.3 | 0.677 | <0.001 | 0.243 |
|  | (0; 56.3) | (25; 81.3) | (31.3; 87.5) | (12.5; 50) | (25; 62.5) | (37.5; 68.8) |  |  |  |
| *Memory and Thinking* | 100 | 96.4 | 100 | 100 | 96.4 | 100 | 0.890 | 0.236 | 0.912 |
|  | (71.4; 100) | (60.7; 100) | (85.7; 100) | (67.9; 100) | (60.7; 100) | (71.4; 100) |  |  |  |
| *Emotion* | 77.8 | 83.3 | 88.9 | 83.3 | 83.3 | 80.6 | 0.818 | 0.520 | 0.192 |
|  | (69.4; 83.3) | (69.4; 91.7) | (75; 94.4) | (58.3; 83.3) | (69.4; 88.9) | (63.9; 86.1) |  |  |  |
| *Communication* | 85.7 | 96.4 | 100 | 100 | 100 | 100 | 0.642 | 0.024 | 0.947 |
|  | (57.1; 96.4) | (53.6; 100) | (85.7; 100) | (78.6; 100) | (85.7; 100) | (96.4; 100) |  |  |  |
| *Activity of daily living* | 37.5 | 47.5 | 47.5 | 37.5 | 42.5 | 60 | 0.321 | <0.001 | 0.003 |
|  | (30; 67.5) | (32.5; 87.5) | (35; 90) | (32.5; 45) | (35; 65) | (42.5; 75) |  |  |  |
| *Mobility* | 42.2 | 61.1 | 75 | 27.8 | 41.7 | 63.9 | 0.467 | <0.001 | 0.290 |
|  | (13.9; 83.3) | (16.7; 94.4) | (30.6; 91.7) | (16.7; 47.2) | (22.2; 72.2) | (52.8; 88.9) |  |  |  |
| *Hand function* | 0 | 0 | 25 | 0 | 0 | 5 | <0.001 | <0.001 | <0.001 |
|  | (0; 50) | (0; 70) | (0; 75) | (0; 0) | (0; 30) | (0; 50) |  |  |  |
| *Social Participation* | 37.5 | 56.3 | 75 | 62.5 | 56.3 | 68.8 | 0.685 | 0.009 | 0.231 |
|  | (28.2; 75) | (37.5; 81.3) | (37.5; 87.5) | (46.9; 71.9) | (28.1; 68.8) | (50; 75) |  |  |  |
| *Recovery* | 50 | 70 | 70 | 40 | 50 | 60 | 0.007 | <0.001 | <0.001 |
|  | (20; 60) | (10; 80) | (10; 90) | (10; 60) | (30; 60) | (30; 80) |  |  |  |
| *Physical* | 30.2 | 35 | 55 | 24.5 | 34 | 51.9 | 0.343 | <0.001 | <0.001 |
|  | (13.4; 67.2) | (22.9; 82.8) | (30.2; 73.9) | (19.9; 37.7) | (27; 52.9) | (48.4; 66.2) |  |  |  |
| Pittsburgh Sleep Quality Index | 8 | 6 | 4 | 6 | 6 | 7 | 0.635 | 0.003 | 0.201 |
|  | (4; 9) | (4; 8) | (4; 9) | (4; 12) | (3; 8) | (4; 10) |  |  |  |

Table S6. Stroke Impact Scale (SIS). Post-hoc, intention-to-treat analysis

| SIS Domains | Group/Time | Comparison^£^ | | Mean  difference | SE | P-value | CI (95%) | |
| --- | --- | --- | --- | --- | --- | --- | --- | --- |
|  |  |  |  |  |  |  | Inferior | Superior |
| Strength | Active/Sham | Pre | Post | -12.58 | 2.15 | <0.001 | -17.73 | -7.44 |
|  |  | Post | Post_3m_ | -6.26 | 2.07 | 0.007 | -11.21 | -1.30 |
| Activity of  daily living | Active | Pre | Post | -9.50 | 1.58 | <0.001 | -14.14 | -4.86 |
|  |  | Post | Post_3m_ | -2.50 | 1.58 | >0.999 | -7.14 | 2.14 |
|  | Sham | Pre | Post | -7.33 | 1.58 | <0.001 | -11.97 | -2.70 |
|  |  | Post | Post_3m_ | -10.00 | 1.58 | <0.001 | -14.64 | -5.36 |
|  | Pre | Active | Sham | 9.33 | 8.62 | >0.999 | -15.95 | 34.62 |
|  | Post | Active | Sham | 11.50 | 8.62 | >0.999 | -13.79 | 36.79 |
|  | Post_3m_ | Active | Sham | 4.00 | 8,62 | >0.999 | -21.29 | 29,29 |
| Mobility | Active/Sham | Pre | Post | -10.74 | 2.47 | <0.001 | -16.66 | -4.82 |
|  |  | Post | Post_3m_ | -10.62 | 2.45 | <0.001 | -16.47 | -4.77 |
| Hand function | Active | Pre | Post | -12.67 | 0.97 | <0.001 | -15.51 | -9.82 |
|  |  | Post | Post_3m_ | -4.67 | 1.07 | <0.001 | -7.82 | -1.52 |
|  | Sham | Pre | Post | -11.33 | 0.75 | <0.001 | -13.53 | -9.14 |
|  |  | Post | Post_3m_ | -14.00 | 0.87 | <0.001 | -16.55 | -11.45 |
|  | Pre | Active | Sham | 19.33 | 1.17 | <0.001 | 15.89 | 22.78 |
|  | Post | Active | Sham | 20.67 | 1.73 | <0.001 | 15.60 | 25.73 |
|  | Post_3m_ | Active | Sham | 11.33 | 2.06 | <0.001 | 5.30 | 17.36 |
| Physical | Active | Pre | Post | -9.86 | 1.31 | <0.001 | -13.71 | -6.01 |
|  |  | Post | Post_3m_ | -0.26 | 1.31 | >0.999 | -4.11 | 3.59 |
|  | Sham | Pre | Post | -11.73 | 1.31 | <0.001 | -15.57 | -7.88 |
|  |  | Post | Post_3m_ | -12.21 | 1.31 | <0.001 | -16.06 | -8.36 |
|  | Pre | Active | Sham | 12.52 | 8.56 | >0.999 | -12.60 | 37.64 |
|  | Post | Active | Sham | 10.65 | 8.56 | >0.999 | -14.47 | 35.78 |
|  | Post_3m_ | Active | Sham | -1.29 | 8.56 | >0.999 | -26.41 | 23.83 |
| Communication | Active/Sham | Pre | Post | -7.77 | 3.34 | 0.060 | -15.76 | 0.23 |
|  |  | Post | Post_3m_ | -4.02 | 3.34 | 0.686 | -12.02 | 3.98 |
| Social Participation | Active/Sham | Pre | Post | -1.47 | 3.48 | >0.999 | -9.81 | 6.86 |
|  |  | Post | Post_3m_ | -10.31 | 3.44 | 0.008 | -18.54 | -2.08 |
| Recovery | Active | Pre | Post | -8.00 | 1.45 | <0.001 | -12.24 | 0.68 |
|  |  | Post | Post_3m_ | -4.00 | 1.52 | 0.130 | -8.47 | 0.63 |
|  | Sham | Pre | Post | -1.33 | 1.33 | >0.999 | -5.24 | -3.76 |
|  |  | Post | Post_3m_ | -11.33 | 1.44 | <0.001 | -15.55 | 0.47 |
|  | Pre | Active | Sham | 4.00 | 2.44 | >0.999 | -3.16 | 2.58 |
|  | Post | Active | Sham | 10.67 | 2.57 | <0.001 | 3.14 | -7.12 |
|  | Post_3m_ | Active | Sham | 3.33 | 2.76 | >0.999 | -4.76 | 11.16 |

CI: confidence interval. £: Comparison is performed between two different time intervals: before treatment compared to after treatment (Pre- Post), and post-treatment compared to 3 months after treatment (Post– Post3m) for each group. We also compared active minus sham values at pre, post and post_3m_ moments for different domains.

Table S7. Stroke Impact Scale (SIS) and Pittsburgh Sleep Quality Index assessed before the first session of treatment (Pre), after the last session of treatment (Post) and three months later (Post_3m_): per-protocol analysis, Generalized Estimating Equation model. Median and interquartile ranges are given

| Outcome | Active | | | | | | | Sham | | | | | | P-value | | | |
| --- | --- | --- | --- | --- | --- | --- | --- | --- | --- | --- | --- | --- | --- | --- | --- | --- | --- |
|  | Pre | | | Post | | Post_3m_ | | Pre | | Post | | Post_3m_ | | Group | Time | Interaction | |
| SIS Domains | |  |  | |  | |  | |  | |  | |  | | | |  |
| *Strength* | 50 | | | 62.5 | | 75 | | 28.1 | | 59.4 | | 62.5 | | 0.181 | <0.001 | 0.16 | |
|  | (31.3; 78.1) | | | (37.5; 90.6) | | (46.9; 90.6) | | (20.3; 46.9) | | (32.8; 62.5) | | (40.6; 73.4) | |  |  |  |  |
| *Memory and Thinking* | 100 | | | 96.4 | | 100 | | 98,2 | | 96.4 | | 100 | | 0.91 | 0.119 | 0.674 | |
|  | (55.4; 100) | | | (67.9; 98.2) | | (85.7; 100) | | (71.4; 100) | | (68.7; 100) | | (76.8; 100) | |  |  |  |  |
| *Emotion* | 80.6 | | | 83.3 | | 88.9 | | 83.3 | | 83.3 | | 79.2 | | 0.493 | 0.772 | 0.214 | |
|  | (70.8; 86.1) | | | (72.2; 88.9) | | (76.4; 91.7) | | (62.5; 83.3) | | (72.2; 91) | | (65.3; 86.1) | |  |  |  |  |
| *Communication* | 82.1 | | | 96.4 | | 100 | | 98.2 | | 100 | | 100 | | 0.653 | 0.058 | 0.975 | |
|  | (64.3; 96.4) | | | (69.7; 100) | | (91.1; 100) | | (81.3; 100) | | (87.5; 100) | | (96.4; 100) | |  |  |  |  |
| *Activities of daily living* | 67.5 | | | 87.5 | | 82.5 | | 37.5 | | 50 | | 62.5 | | 0.208 | <0.001 | <0.001 | |
|  | (22.5; 75) | | | (31.3; 95) | | (38.8; 98.8) | | (33.1; 48.8) | | (35.6; 66.9) | | (47.5; 76.9) | |  |  |  |  |
| *Mobility* | 77.8 | | | 94.4 | | 91,7 | | 38.9 | | 50 | | 73.6 | | 0.309 | <0.001 | 0.309 | |
|  | (18.1;98.6) | | | (23.6; 98.6) | | (75; 100) | | (19.4; 63.9) | | (37.5; 80.6) | | (59; 88.9) | |  |  |  |  |
| *Hand function* | 20 | | | 50 | | 65 | | 0 | | 2.5 | | 20 | | <0.001 | <0.001 | <0.001 | |
|  | (0; 50) | | | (0; 85) | | (12.5; 95) | | (0; 0) | | (0; 33.8) | | (0; 68.8) | |  |  |  |  |
| *Social Participation* | 36.9 | | | 65.6 | | 78,1 | | 65.5 | | 57.8 | | 70.3 | | 0.894 | 0.005 | 0.154 | |
|  | (26.6;68.8) | | | (18.8; 89.1) | | (54.7; 100) | | (50; 71.1) | | (31.3; 68) | | (51.6; 75) | |  |  |  |  |
| *Recovery* | 50 | | | 70 | | 70 | | 45 | | 50 | | 65 | | <0.001 | <0.001 | 0.01 | |
|  | (40; 60) | | | (50; 80) | | (70; 85) | | (17.5; 57.5) | | (40; 60) | | (50; 80) | |  |  |  |  |
| *Physical* | 54.6 | | | 72.4 | | 69.4 | | 25.9 | | 47.4 | | 59.3 | | 0.116 | <0.001 | <0.001 | |
|  | (18; 71.7) | | | (23.1; 90.4) | | (47.1; 74.5) | | (20.3; 39.5) | | (28.9; 55.8) | | (49.5; 67.1) | |  |  |  |  |
| Pittsburgh Sleep Quality Index | 7 | | | 6 | | 4 | | 5 | | 5.5 | | 7 | | 0.736 | 0.053 | 0.232 | |
|  | (4.5;8) | | | (4.5;7) | | (4;8) | | (4;8) | | (4;8) | | (4;10) | |  |  |  | |

Table S8. Stroke Impact Scale (SIS). Post-hoc, per-protocol analysis

| Scale | Group/Time | Comparison^£^ | | Mean difference | SE | P- Value | IC (95%) | |
| --- | --- | --- | --- | --- | --- | --- | --- | --- |
| SIS Domains |  |  |  |  |  |  | Inferior | Superior |
| Strength | Active and Sham | Pre | Post | -15.71 | 2.71 | <0.001 | -22.2 | -9.21 |
|  |  | Post | Post_3m_ | -8.89 | 2.61 | 0.002 | -15.13 | -2.64 |
| Activities of  daily living | Active | Pre | Post | -13.61 | 1.13 | <0.001 | -16.92 | -10.3 |
|  |  | Post | Post_3m_ | -4.17 | 1.13 | 0.003 | -7.48 | -0.86 |
|  | Sham | Pre | Post | -10.21 | 0.98 | <0.001 | -13.07 | -7.34 |
|  |  | Post | Post_3m_ | -12.5 | 0.98 | <0.001 | -15.37 | -9.63 |
|  | Pre | Active | Sham | 13.33 | 10.41 | >0.999 | -17.21 | 43.88 |
|  | Post | Active | Sham | 16.74 | 10.41 | >0.999 | -13.81 | 47.28 |
|  | Post_3m_ | Active | Sham | 8.4 | 10.41 | >0.999 | -22.14 | 38.95 |
| Mobility | Active and Sham | Pre | Post | -11.83 | 2.82 | <0.001 | -18.58 | -5.08 |
|  |  | Post | Post_3m_ | -14.82 | 2.79 | <0.001 | -21.5 | -8.14 |
| Hand function | Active | Pre | Post | -21.11 | 1.58 | <0.001 | -25.75 | -16.48 |
|  |  | Post | Post_3m_ | -7.78 | 1.78 | <0.001 | -13.01 | -2.55 |
|  | Sham | Pre | Post | -14.17 | 1.09 | <0.001 | -17.36 | -10.98 |
|  |  | Post | Post_3m_ | -17.5 | 1.14 | <0.001 | -20.83 | -14.17 |
|  | Pre | Active | Sham | 25 | 1.67 | <0.001 | 20.11 | 29.89 |
|  | Post | Active | Sham | 31.94 | 2.51 | <0.001 | 24.57 | 39.31 |
|  | Post_3m_ | Active | Sham | 22.22 | 2.94 | <0.001 | 13.6 | 30.84 |
| Physical | Active | Pre | Post | -13.69 | 1.21 | <0.001 | -17.23 | -10.16 |
|  |  | Post | Post_3m_ | -0.44 | 1.21 | >0.999 | -3.98 | 3.1 |
|  | Sham | Pre | Post | -14.25 | 1.04 | <0.001 | -17.32 | -11.19 |
|  |  | Post | Post_3m_ | -15.26 | 1.04 | <0.001 | -18.33 | -12.2 |
|  | Pre | Active | Sham | 18.43 | 8.97 | 0.598 | -7.89 | 44.76 |
|  | Post | Active | Sham | 17.88 | 8.97 | 0.694 | -8.45 | 44.2 |
|  | Post_3m_ | Active | Sham | 3.05 | 8.97 | >0.999 | -23.28 | 29.38 |
| Social Participation | Active and Sham | Pre | Post | -2.73 | 4.89 | >0.999 | -14.44 | 8.97 |
|  |  | Post | Post_3m_ | -14.99 | 4.74 | 0.005 | -26.34 | -3.65 |
| Recovery | Active | Pre | Post | -12.22 | 2.93 | <0.001 | -20.83 | -3.62 |
|  |  | Post | Post_3m_ | -6.67 | 3.16 | 0.524 | -15.94 | 2.61 |
|  | Sham | Pre | Post | -1.67 | 2.25 | >0.999 | -8.27 | 4.93 |
|  |  | Post | Post_3m_ | -14.17 | 2.44 | <0.001 | -21.34 | -6.99 |
|  | Pre | Active | Sham | 6.94 | 3.06 | 0.348 | -2.04 | 15.92 |
|  | Post | Active | Sham | 17.5 | 3.3 | <0.001 | 7.83 | 27.17 |
|  | Post_3m_ | Active | Sham | 10 | 3.58 | 0.077 | -0.49 | 20.49 |

CI: confidence interval. £: Comparison is performed between two different time intervals: before treatment compared to after treatment (Pre- Post), and post-treatment compared to 3 months after treatment (Post– Post3m) for each group. We also compared active minus sham values at pre, post and post_3m_ moments for different domains.

Table S9. Generalized Estimating Equations, intention-to-treat (ITT) and per-protocol analyses of Fugl-Meyer Assessment (FMA) scores, adjusted for pre-treatment FMA scores (covariate)

| Factor | Test statistic (Wald)  ITT | P-value  ITT | Test statistic (Wald)  Per protocol | P-value  Per protocol |
| --- | --- | --- | --- | --- |
| FMA (pre-treatment) | 204.77 | <0.001 | 204.77 | <0.001 |
| Group | 13.40 | <0.001 | 9.73 | 0.002 |
| Time | 4.99 | 0.026 | 4.01 | 0.045 |
| Group*Time | 0.38 | 0.535 | 0.02 | 0.896 |
|  |  |  |  |  |

Table S10. Post-hoc intention-to-treat and per-protocol analyses of Fugl-Meyer Assessment (FMA) scores, adjusted for baseline FMA scores (covariate)

| Comparison | Mean difference  between FMA scores | Standard error | P-value | Confidence Interval (95%) | |
| --- | --- | --- | --- | --- | --- |
|  |  |  |  | Inferior | Superior |
| Intention to treat |  |  |  |  |  |
| Group (Active *versus* Sham) | -8.01 | 2.57 | 0.002 | -13.0 | -2.98 |
| Time (Post *versus* 3 months) | -1.93 | 0.96 | 0.045 | -3.82 | -0.04 |
|  |  |  |  |  |  |
| Per-protocol |  |  |  |  |  |
| Group (Active *versus* Sham) | -6.83 | 1.87 | <0.001 | -10.5 | -3.17 |
| Time (Post *versus* 3 months) | -1.15 | 0.51 | 0.026 | -3.82 | -0.14 |

Table S11. Minimal clinically important difference. Per-protocol analysis of changes before and after treatment (Pre-Post), and post-treatment compared to 3 months after treatment (Post-Post_3m_)

| Outcome | Active | | Sham | | P-value | | |
| --- | --- | --- | --- | --- | --- | --- | --- |
|  | Pre – Post | Post - Post_3m_ | Pre - Post | Post - Post_3m_ | Group | Time | Interaction |
|  | n (%) | n (%) | n (%) | n (%) |  |  |  |
| Fugl-Meyer Assessment | 4 (44.4) | 0 (0) | 7 (63.6) | 0 (0) | 0.739 | 0.005 | 0.573 |
| Motor Activity Log, qualitative | 5 (55.6) | 0 (0) | 4 (36.4) | 3 (27.3) | 0.71 | 0.04 | 0.078 |
| National Institutes of Health Stroke Scale | 1 (11.1) | 0 (0) | 2 (18.2) | 2 (18.2) | 0.311 | 0.808 | 0.823 |
| Modified Rankin Scale | 4 (44.4) | 4 (44.4) | 6 (54.5) | 4 (36.4) | 0.942 | 0.633 | 0.633 |
| Barthel index | 1 (11.1) | 0 (0) | 2 (18.2) | 1 (9,1) | 0.506 | 0.959 | 0.988 |

**Summary of the Study Protocol approved by the Ethics Comittee**

**Introduction**

Stroke is a major cause of disability worldwide. Upper limb paresis occurs in more than 80% of the patients and has substantial impact on performance of daily activities [1]. Transcranial direct current stimulation (tDCS) has emerged as a potential strategy to decrease disability associated with arm and hand impairments [2-7].

According to the hypothesis of interhemispheric inhibition, the unaffected motor cortex may excessively inhibit the motor cortex of the affected hemisphere in subjects with stroke and contralateral upper limb paresis [8]. tDCS can be used to either inhibit the unaffected hemisphere, excite the affected hemisphere, or both [9]. Typically, anodal tDCS increases brain excitability and cathodal tDCS has the opposite effect, but these results can vary according to cortical excitability [10].

A systematic review and metanalysis underscored the lack of information about adverse events of tDCS [11]. Even though no serious adverse events of tDCS have been reported [12], few studies provided detailed information about tolerability and safety of this intervention [13]. Adverse events were described as mild and led to the drop-out of only one subject after cathodal tDCS of the unaffected motor cortex in only one study performed in the early phase after stroke, but structured questionnaires were not applied in this or other studies [14].

Excessive excitation of the affected hemisphere, either directly by ipsilesional anodal tDCS or indirectly by contralesional cathodal tDCS might be harmful, as suggested by findings in rats of increased infarct size after anodal tDCS [15], and decreased cerebral blood flow after cathodal tDCS [16, 17]. Also, tDCS of the motor cortex may affect other brain areas [18], leading to non-motor effects on blood pressure, heart rate and cognitive function [19-25]. Such effects had yet not been investigated in subjects with stroke.

**Study goals and objectives**

The primary goal of this study is to compare safety and tolerability of six sessions of active cathodal or sham tDCS of the unaffected motor cortex (ctDCS_UH_) in subjects at an early phase after stroke, as add-on interventions to physical therapy. The secondary goal is to preliminarily evaluate efficacy of the intervention between both groups.

Our specific primary objective is to compare the frequency of adverse events in the active group versus sham group.

Moreover, as secondary specific objectives, we will evaluate systolic, diastolic and mean blood pressure measurements before and after each tDCS session.

Additionally, we will compare preliminary measurements of efficacy between active versus sham ctDCS_UH_ group on motor performance, use of the paretic upper limb in activities of daily living, spasticity, overall neurological impairment, disability, quality of life, cognition and sleep quality. These outcomes will be compared before, immediately after treatment and three months later. Ultimately, stroke lesion volumes and recurrence will be evaluated by MRI before and after treatment.

**Study Design**

The study is a randomized two-arm, double-blind, sham-controlled clinical trial that will be performed at the Albert Einstein Hospital until follow-up of the last subject. The protocol was approved by the Albert Einstein Hospital´s Ethics Committee. The protocol was registered at Clinicaltrials.gov (NCT 024555427). The research will be conducted according to standards of the declaration of Helsinki and Brazilian regulations (Resolution CNS 466/12) [26].

Informed consent will be required from all patients. However, for those not able to sign we will allow proxies to provide consent.

Inclusion criteria: Age ≥18 years; ischemic stroke at least 72 hours and up to 6 weeks before enrollment, confirmed by CT or MRI; upper limb paresis defined as a minimum score of 1 in subitem 5a or 5b of the National Institute of Health Stroke Scale (NIHSS) [27]. We will include subjects in the acute (up to 7 days) and early subacute (from 7 days to 3 months) phases after stroke [28].

Exclusion Criteria: advanced systemic disease; clinical instability such as uncontrolled cardiac arrhythmia or heart failure; dementia; history of prior stroke affecting the corticospinal tract of the contralateral M1; strokes affecting the cerebellum or cerebellar pathways; contraindications to tDCS; Modified Rankin Scale >2 prior to stroke; pregnancy; contraindication for physical therapy; comprehension aphasia [11,12,29,30].

**Baseline measures**

The following baseline variables will be collected: age; gender; race; education; side and site of lesion classified into cortical, cortico-subcortical and if it includes motor primary cortex and/or internal capsule; vascular risk factors (arterial hypertension, diabetes mellitus, history of prior stroke and current smoker status); alcohol use; handedness according to Edinburgh Handedness Inventory [31]; conditions related to the recent stroke such as thrombolysis; time since stroke; depression or anxiety scored according to the Hospital Anxiety and Depression Scale (HADS) [32] and some baseline scores will be applied as Trial of Org 10172 in Acute Stroke Treatment (TOAST) score [33].

**Experimental protocol**

***Enrollment, randomization and blinding***

Recruitment will be performed at Hospital Albert Einstein and in the community [34]. We will create a computer-generated blocked-randomization schedule (10 blocks of 4 subjects) with *randomization.com* for allocation to either the active or sham tDCS group at a 1:1 ratio.

We will keep the randomization table in a locked cabinet and in password-protected files, accessible only to the investigator who will administer tDCS and the principal investigator. Patients and researchers responsible for administering physical therapy or evaluating outcomes will not be aware of group assignment. After the last tDCS session, a blinded investigator will ask all patients whether they believed they received real stimulation or not.

***Intervention***

Patients will undergo 3 sessions of treatment per week, every other day from Monday to Friday for 2 weeks, in a total of 6 sessions. In each session, a rubber sponge electrode (7x5 cm) soaked in saline solution will be placed over the ipsilesional supraorbital area (anodal electrode) and fixed by a nonconducting, nonabsorbent elastic strap. Other electrode (cathode) will be placed on the contralesional C3/C4 position according to the EEG 10-20 reference system [35]. The intensity of stimulation will be 1mA, ramps up and down will last for 10 seconds (DC-stimulator plus, Neuroconn, Germany). In the active group, tDCS will be applied for 20 minutes and in the sham group, for 30 seconds. This sham set-up reduces bias from unblinding [3, 36-38].

Before turning on the tDCS device, safety data regarding vital signs will be collected using an automatic device: arterial blood pressure (systolic, diastolic and mean blood pressure) and heart rate. Right after finishing stimulation, the same parameters will be collected for comparison.

Two non-blinded neurologists will be allowed to apply tDCS, according to the allocation group. Considering that hyperemia is a relatively common skin reaction in patients that receive the real stimulation, before the questionnaire is applied by a blinded member for the group allocation, the frontal region of the head, where the anode electrode is placed, will be covered with the objective to hide a possible hyperemia.

A blinded member will apply a structured questionnaire. Once the non-invasive stimulation session and the questionnaire assessment are finished, participants will receive a 50-minute session of physiotherapy with passive and active exercises for upper and lower limbs, focusing on the upper extremity.

After the end of the 6 sessions, the patient will be asked by the same investigator to guess if the stimulation was real or not, with additional explanation about the reasons of the negative or positive answer.

After collecting data about safety, a blinded physical therapist will start a conventional session of physical therapy for a total of 50 minutes with 30 min focused on upper limb. The participant will perform exercises selected from a standard program, including passive and active movements, focused on strength improvement, targeting upper limb and adapted to their individual needs. Blood arterial pressure will be measured before and after the physical therapy session

The numbers of hours of physical therapy performed outside of the protocol, during treatment and for the next three months after the end of treatment will be registered according to information provided by the patients.

**Assessments**

All subjects will be evaluated by the researcher with respect to safety (primary outcome measures and secondary outcome - neuroimaging measures) and efficacy (secondary outcome - clinical measures).

For primary safety measures, patients will be assessed before and right after each session. For efficacy, patients will be assessed at three time-points: before the first session of treatment (pre), after the last session of treatment (post) and 3 months after end of treatment (Post_3m_). For other secondary measures (neuroimaging), individuals were assessed at two time-points: pre- and post-stimulation.

***Primary outcome measures***

The safety primary outcomes will be measured by the frequency of adverse events and changes in vital signs. The adverse events will be reported spontaneously, during tDCS session, after a structured questionnaire and will be observed by the researcher.

***1) Spontaneous report during tDCS, observed adverse events and questionnaire report***.

During tDCS, the neurologist will ask systematically (each 5 minutes) the patient if he/she would like to report anything. If an adverse event is reported, the unblinded neurologist will register it on the Clinical Report Form. Serious adverse events will be reported to the Ethics Committee within 24 hours.

After the tDCS session, an allocation-blinded researcher will apply a safety questionnaire proposed by Fertonani et al [39]. This tool is comprised of open questions (“Is there any adverse reaction that you feel during stimulation?”) but also of direct questions (“Did you feel itching sensation during stimulation?”) about specific symptoms already reported by other individual in the literature (fatigue, itching, tingling, burning, pinching, heat, pain, metallic or iron taste, lack of concentration, visual disturbances, dizziness, anxiety, sleepiness or other symptoms). Also, the researcher will score the intensity of the adverse reaction using a Visual Analogic Scale (VAS) and will assess the duration, as well as its temporal relationship with the tDCS stimulation [40].

***2) Vital Signs***. At each stimulation session, blood pressure and heart rate will be measured twice (before starting tDCS and few minutes after the end of intervention) with an automated sphygmomanometer (BP3BTO-A, Microlife). The vital signs will be measured when the patient is comfortable, sitting, for at least 10 minutes.

***Secondary outcomes***

***Clinical outcomes***

The following outcomes will be assessed before the first session of treatment (Pre), after the last session of treatment (Post) and three months later (Post_3m_): upper limb motor impairment, subitem 5a or 5b of National Institute of Health Stroke Scale (NIHSS_5_) [27] and Fugl-Meyer Assessment of Motor Recovery after Stroke (FMA) [41]; upper limb use in daily living, Motor Activity Log (MAL) [42]; upper limb spasticity, Modified Ashworth Scale (MAS) [43]; overall neurologic impairment, National Institute of Health Stroke Scale total score (NIHSS_total_) [27]; overall disability, modified Rankin Scale [27,44]; functional independence, Barthel Index (BI) [27]; quality of life, Stroke Impact Scale (SIS) [45]; cognition, Montreal Cognitive Assessment (MoCA) [46] and Sleep Quality Index (PSQI) questionnaire [47].

National Institute of Health Stroke Scale (NIHSS) [27] – this 15-item scale, with a total score of 42, is a reliable predictor of the severity of stroke by assessing standardized items of neurological examination. This score is widely used in clinical practice and research, to assess global neurological impairment. We will also adopt the motor item (5a or 5b) to quantify the motor strength for upper limbs. We use the translated and adapted NIHSS Portuguese version.

Fugl-Meyer Assessment Upper Extremity [41] – this scale aims to evaluate the motor impairment after stroke in clinical and research. It has good intra- and inter- observer reliabilities and will be used to assess the recovery of our subjects at the three time-points (pre, post and Post_3m_). We will use the Upper Extremity (UE) subscale for quantifying the level of disability of upper limb related to motor performance (33 items, score range 0-66).

Motor Activity Log (MAL) [42] – we will use this scale to evaluate spontaneous motor activity with the most impairment upper limb performing daily living activities. We used a standard questionnaire of 30 items asking the patients qualitatively (quality of movement) and quantitatively (the amount of use), about their capacity to do activities with the affected hand.

Modified Ashworth Scale (MAS) [43]– this clinical scale was implemented to evaluate the spasticity rate of upper extremity muscles at the level of shoulder, elbow, wrist and fingers. Spasticity is manually measured by passively stretching the muscle through its range of motion and graduating the resistance from 0 to 4.

Modified Rankin Scale (mRS) [27,44]– we will assess global outcome after stroke by applying this widely used scale. This scale has 6 different levels of disability were grade 5 is severe disability and grade 0 is normal individual.

Barthel Index (BI) [27] – this scale is used to evaluate functional outcome after stroke. It comprises 10 items that evaluate basic activities of daily living that whether performed, demonstrates the level of independence of the patient. The final score is categorized in 5 levels of dependency (range of score 0-100 points, total dependency 0-20; severe dependency 21-40; moderate dependency 41-60, mild dependency 61-90 and total independence 91-100). This index has excellent psychometric qualities and inter-rater reliability.

Stroke Impact Scale (SIS) [45] – this scale provides information about the perceptions of the patients and caregivers about the stroke. The SIS 3.0 comprises 59 questions about health-related quality of life, and 8 domains as follow: strength, hand function, mobility, physical and instrumental activities of daily living, memory and thinking, communication, emotion and social participation. Each item is rated from 0-100 and higher scores reflect higher quality of life.

Montreal Cognitive Assessment (MOCA) [46] – we will assess cognitive impairment by using MOCA-test Brazilian version. This test, with a range of 0-30 points, has high sensibility for detecting early-stage cognitive impairment compared to the Mini-mental State Examination (MMSE).

Pittsburgh Sleep Quality Index ( PSQI) [47] questionnaire – we will assess the quality of sleep by applying a structured interview about 7 components (scored from 0 to 3 with global score that ranges from 0- 21): subjective sleep quality, sleep latency, sleep duration, habitual sleep efficiency, sleep disturbances, use of sleeping medication and daytime dysfunction. Higher scores reflect poor sleep quality with a cut-off score of 5.

Baseline measures will be assessed for differences between groups using Chi-square tests for categorical variables and unpaired t-tests, or Mann-Whitney tests for continuous variables according to distribution of the data.

Chi-square tests will be used to compare drop-out rates, frequencies of blinding to the tDCS condition, as well as primary safety outcomes between the active and sham groups. The frequency of adverse events will be reported only for subjects submitted to at least one session of tDCS.

Secondary clinical outcomes will be analyzed with Generalized Estimating Equations (GEE) with factors time (pre-intervention, post-intervention and after 3 months) and group (active or sham). Post-hoc analyses will be performed with Bonferroni´s correction for multiple comparisons.

In addition, we will evaluate Minimal Clinically Important Differences (MCID) of the following outcomes, as described for subjects in the early phase post-stroke: FMA (9 points) [48], qualitative MAL (1 point) [49], NIHSS (3 points) [50, 51], mRS[52] and BI (20 points) [53] and PSQI (3 points) [54].

Intention-to-treat (ITT) and per protocol analyses will be performed. For ITT, missing observations were imputed with the Last Observation Carried Forward (LOCF). In per-protocol analysis, data from patients who completed at least 5 sessions of treatment and all sessions of evaluation of outcomes will be analyzed. Analyses will be conducted with SPSS-software (SPSS 20.0 for Windows; SPSS Inc.). P-values < 0.05 will be considered statistically significant.

**Quality Assurance**

The investigators will conduct the research according to current guidelines of good clinical practice.

An independent review board (Albert Einstein’s Hospital Clinical Research Center) will review clinical research and informed consent forms, every six months.

**Ethics**

Informed consent will be required from all patients. However, for those not able to sign we will allow proxies to provide in writing. The protocol was approved by the Ethics committee of Hospital Israelita Albert Einstein and will be performed in accordance with the ethical standards laid down in the 1964 Declaration of Helsinki and its later amendments.

**References**

1. Dobkin, B.H., Clinical practice. Rehabilitation after stroke. N Engl J Med, 2005. 352(16): p. 1677-84.

2. Kang, N., J.J. Summers, and J.H. Cauraugh, Transcranial direct current stimulation facilitates motor learning post-stroke: a systematic review and meta-analysis. J Neurol Neurosurg Psychiatry, 2015.

3. Adeyemo, B.O., et al., Systematic review of parameters of stimulation, clinical trial design characteristics, and motor outcomes in non-invasive brain stimulation in stroke. Front Psychiatry, 2012. 3: p. 88.

4. Butler, A.J., et al., A meta-analysis of the efficacy of anodal transcranial direct current stimulation for upper limb motor recovery in stroke survivors. J Hand Ther, 2013. 26(2): p. 162-70; quiz 171.

5. Chhatbar, P.Y., et al., Transcranial Direct Current Stimulation Post-Stroke Upper Extremity Motor Recovery Studies Exhibit a Dose-Response Relationship. Brain Stimul, 2016. 9(1): p. 16-26.

6. Bastani, A. and S. Jaberzadeh, Does anodal transcranial direct current stimulation enhance excitability of the motor cortex and motor function in healthy individuals and subjects with stroke: a systematic review and meta-analysis. Clin Neurophysiol, 2012. 123(4): p. 644-57.

7. Elsner, B., et al., Transcranial direct current stimulation (tDCS) for improving activities of daily living, and physical and cognitive functioning, in people after stroke. Cochrane Database Syst Rev, 2016. 3: p. Cd009645.

8. Nowak, D.A., et al., Interhemispheric competition after stroke: brain stimulation to enhance recovery of function of the affected hand. Neurorehabil Neural Repair, 2009. 23(7): p. 641-56.

9. Schlaug, G., V. Renga, and D. Nair, Transcranial direct current stimulation in stroke recovery. Arch Neurol, 2008. 65(12): p. 1571-6.

10. Hsu, T.Y., C.H. Juan, and P. Tseng, Individual Differences and State-Dependent Responses in Transcranial Direct Current Stimulation. Front Hum Neurosci, 2016. 10: p. 643.

11. Brunoni, A.R., et al., A systematic review on reporting and assessment of adverse effects associated with transcranial direct current stimulation. Int J Neuropsychopharmacol, 2011. 14(8): p. 1133-45.

12. Bikson, M., et al., Safety of Transcranial Direct Current Stimulation: Evidence Based Update 2016. Brain Stimul, 2016.

13. Russo, C., et al., Safety Review of Transcranial Direct Current Stimulation in Stroke. Neuromodulation, 2017. 20(3): p. 215-222.

14. Kim, D.Y., et al., Effect of transcranial direct current stimulation on motor recovery in patients with subacute stroke. Am J Phys Med Rehabil, 2010. 89(11): p. 879-86.

15. Peruzzotti-Jametti, L., et al., Safety and efficacy of transcranial direct current stimulation in acute experimental ischemic stroke. Stroke, 2013. 44(11): p. 3166-74.

16. Wachter, D., et al., Transcranial direct current stimulation induces polarity-specific changes of cortical blood perfusion in the rat, in Exp Neurol. 2011, A 2010 Elsevier Inc: United States. p. 322-7.

17. Mielke, D., et al., Cathodal transcranial direct current stimulation induces regional, long-lasting reductions of cortical blood flow in rats. Neurol Res, 2013. 35(10): p. 1029-37.

18. Hummel, F.C. and L.G. Cohen, Non-invasive brain stimulation: a new strategy to improve neurorehabilitation after stroke? Lancet Neurol, 2006. 5(8): p. 708-12.

19. Schestatsky, P., et al., Non-invasive brain stimulation and the autonomic nervous system. Clin Neurophysiol, 2013. 124(9): p. 1716-28.

20. Vernieri, F., et al., Cortical neuromodulation modifies cerebral vasomotor reactivity. Stroke, 2010. 41(9): p. 2087-90.

21. Santarnecchi, E., et al., Time Course of Corticospinal Excitability and Autonomic Function Interplay during and Following Monopolar tDCS. Front Psychiatry, 2014. 5: p. 86.

22. Krone, L., et al., Top-down control of arousal and sleep: Fundamentals and clinical implications. Sleep Med Rev, 2017. 31: p. 17-24.

23. Ebajemito, J.K., et al., Application of Transcranial Direct Current Stimulation in Neurorehabilitation: The Modulatory Effect of Sleep. Front Neurol, 2016. 7: p. 54.

24. Au-Yeung, S.S., et al., Transcranial direct current stimulation to primary motor area improves hand dexterity and selective attention in chronic stroke. Am J Phys Med Rehabil, 2014. 93(12): p. 1057-64.

25. Park, J.Y., et al., Significance of longitudinal changes in the default-mode network for cognitive recovery after stroke. Eur J Neurosci, 2014. 40(4): p. 2715-22.

26. Medicina, Conselho Federal. Resolução CFM 1.986/2012 [ Internet]. 2012; Available from: http://www.portalmedico.org.br/resolucoes/CFM/2012/1986_2012.pdf.

27. Cincura, C., et al., Validation of the National Institutes of Health Stroke Scale, modified Rankin Scale and Barthel Index in Brazil: the role of cultural adaptation and structured interviewing. Cerebrovasc Dis, 2009. 27(2): p. 119-22.

28. Bernhardt, J., et al., Early rehabilitation after stroke. Curr Opin Neurol, 2017. 30(1): p. 48-54.

29. Potter-Baker, K.A., et al., Challenges in Recruitment for the Study of Noninvasive Brain Stimulation in Stroke: Lessons from Deep Brain Stimulation. J Stroke Cerebrovasc Dis, 2016.

30. Brunoni, A.R., et al., Clinical research with transcranial direct current stimulation (tDCS): challenges and future directions. Brain Stimul, 2012. 5(3): p. 175-95.

31. Oldfield, R.C., The assessment and analysis of handedness: the Edinburgh inventory. Neuropsychologia, 1971. 9(1): p. 97-113.

32. Zigmond, A.S. and R.P. Snaith, The hospital anxiety and depression scale. Acta Psychiatr Scand, 1983. 67(6): p. 361-70.

33. Adams, H.P., Jr., et al., Design of the Trial of Org 10172 in Acute Stroke Treatment (TOAST). Control Clin Trials, 1997. 18(4): p. 358-77.

34. Pires, D.S. et al., Sucesso de estratégias de divulgação de um protocolo de reabilitação em doenças cerebrovasculares 2017, Centro Universitário das Faculdades Metropolitanas Unidas - FMU.

35. DaSilva, A.F., et al., Electrode positioning and montage in transcranial direct current stimulation. J Vis Exp, 2011(51).

36. Nitsche, M.A., et al., Transcranial direct current stimulation: State of the art 2008. Brain Stimul, 2008. 1(3): p. 206-23.

37. Mahmoudi, H., et al., Transcranial direct current stimulation: electrode montage in stroke. Disabil Rehabil, 2011. 33(15-16): p. 1383-8.

38. Woods, A.J., et al., A technical guide to tDCS, and related non-invasive brain stimulation tools. Clin Neurophysiol, 2016. 127(2): p. 1031-48.

39. Fertonani, A., C. Ferrari, and C. Miniussi, What do you feel if I apply transcranial electric stimulation? Safety, sensations and secondary induced effects. Clin Neurophysiol, 2015. 126(11): p. 2181-8.

40. Gandiga, P.C., F.C. Hummel, and L.G. Cohen, Transcranial DC stimulation (tDCS): a tool for double-blind sham-controlled clinical studies in brain stimulation. Clin Neurophysiol, 2006. 117(4): p. 845-50.

41. Sullivan, K.J., et al., Fugl-Meyer assessment of sensorimotor function after stroke: standardized training procedure for clinical practice and clinical trials. Stroke, 2011. 42(2): p. 427-32.

42. de Caneda, M.A., et al., [Reliability of neurological assessment scales in patients with stroke]. Arq Neuropsiquiatr, 2006. 64(3a): p. 690-7.

43. Bohannon, R.W. and M.B. Smith, Interrater reliability of a modified Ashworth scale of muscle spasticity. Phys Ther, 1987. 67(2): p. 206-7.

44. Bonita, R. and R. Beaglehole, Recovery of motor function after stroke. Stroke, 1988. 19(12): p. 1497-500.

45. Carod-Artal, F.J., et al., The stroke impact scale 3.0: evaluation of acceptability, reliability, and validity of the Brazilian version. Stroke, 2008. 39(9): p. 2477-84.

46. Memoria, C.M., et al., Brief screening for mild cognitive impairment: validation of the Brazilian version of the Montreal cognitive assessment. Int J Geriatr Psychiatry, 2013. 28(1): p. 34-40.

47. Buysse, D.J., et al., The Pittsburgh Sleep Quality Index: a new instrument for psychiatric practice and research. Psychiatry Res, 1989. 28(2): p. 193-213.

48. Arya KN, Verma R, Garg RK. Estimating the minimal clinically important difference of an upper extremity recovery measure in subacute stroke patients. Top Stroke Rehabil. 2011;18 Suppl 1:599-610.

49. Lang CE, Edwards DF, Birkenmeier RL, Dromerick AW. Estimating minimal clinically important differences of upper extremity measures early after stroke. Archives of physical medicine and rehabilitation. 2008;89(9):1693.

50. Adams HP, Jr., Davis PH, Leira EC, Chang KC, Bendixen BH, Clarke WR, et al. Baseline NIH Stroke Scale score strongly predicts outcome after stroke: A report of the Trial of Org 10172 in Acute Stroke Treatment (TOAST). Neurology. 1999;53(1):126-31.

51. Harrison JK, McArthur KS, Quinn TJ. Assessment scales in stroke: clinimetric and clinical considerations. Clin Interv Aging. 2013;8:201-11.

52. Banks JL, Marotta CA. Outcomes validity and reliability of the modified Rankin scale: implications for stroke clinical trials: a literature review and synthesis. Stroke. 2007;38(3):1091-6.

53. Quinn TJ, Langhorne P, Stott DJ. Barthel index for stroke trials: development, properties, and application. Stroke. 2011;42(4):1146-51.

54. Hughes CM, McCullough CA, Bradbury I, Boyde C, Hume D, Yuan J, et al. Acupuncture and reflexology for insomnia: a feasibility study. Acupunct Med. 2009;27(4):163-8.
